# Glycogen Metabolic Genes Are Involved in Trehalose-6-Phosphate Synthase-Mediated Regulation of Pathogenicity by the Rice Blast Fungus *Magnaporthe oryzae*

**DOI:** 10.1371/journal.ppat.1003604

**Published:** 2013-10-03

**Authors:** Muhammad Badaruddin, Lucy J. Holcombe, Richard A. Wilson, Zheng-Yi Wang, Michael J. Kershaw, Nicholas J. Talbot

**Affiliations:** School of Biosciences, University of Exeter, Geoffrey Pope Building, Exeter, Devon, United Kingdom; Commonwealth Scientific and Industrial Research Organisation (CSIRO), Australia

## Abstract

The filamentous fungus *Magnaporthe oryzae* is the causal agent of rice blast disease. Here we show that glycogen metabolic genes play an important role in plant infection by *M. oryzae*. Targeted deletion of *AGL1* and *GPH1*, which encode amyloglucosidase and glycogen phosphorylase, respectively, prevented mobilisation of glycogen stores during appressorium development and caused a significant reduction in the ability of *M. oryzae* to cause rice blast disease. By contrast, targeted mutation of *GSN1*, which encodes glycogen synthase, significantly reduced the synthesis of intracellular glycogen, but had no effect on fungal pathogenicity. We found that loss of *AGL1* and *GPH1* led to a reduction in expression of *TPS1* and *TPS3*, which encode components of the trehalose-6-phosphate synthase complex, that acts as a genetic switch in *M. oryzae*. Tps1 responds to glucose-6-phosphate levels and the balance of NADP/NADPH to regulate virulence-associated gene expression, in association with Nmr transcriptional inhibitors. We show that deletion of the *NMR3* transcriptional inhibitor gene partially restores virulence to a Δ*agl1Δgph1* mutant, suggesting that glycogen metabolic genes are necessary for operation of the NADPH-dependent genetic switch in *M. oryzae*.

## Author Summary

The fungus *Magnaporthe oryzae* causes a devastating disease of rice called blast. Each year, rice blast disease destroys almost a quarter of the potential global rice harvest. The fungus infects rice plants by elaborating a special infection structure called an appressorium, which physically breaks the tough outer cuticle of a rice leaf. Magnaporthe can develop appressoria in the absence of a nutrient source. We are therefore studying how the fungus utilizes energy stores in its spores to fuel its initial growth and development. Glycogen is a key storage compound in fungi and in this study we have investigated how glycogen breakdown occurs in the rice blast fungus. We have shown that the two major enzymes that degrade cellular stores of glycogen are important in rice blast disease. However, we also found that a strain of the fungus which is severely impaired in its ability to synthesize its own glycogen can still infect plants normally. To explain these apparently contradictory findings we explored the regulatory role of glycogen breakdown and provide evidence that glycogen metabolism is a key regulator of a recently described, virulence-associated genetic switch in Magnaporthe that is operated by an enzyme called trehalose-6-phosphate synthase.

## Introduction

Rice blast disease is the most serious disease of cultivated rice and in recent years has caused epidemics in South Korea, Japan, Bhutan and China [1], [2], resulting in severe harvest losses. Understanding the biology of rice blast disease is therefore important, if durable control strategies for the disease are to be developed [2].

The rice blast fungus, *Magnaporthe oryzae*, brings about infection of rice leaves by forming a specialized infection structure called an appressorium [2]. This is a unicellular, dome-shaped structure that develops from the end of a fungal germ tube and is responsible for mechanically breaching the rice cuticle, allowing the fungus entry to plant cells. Appressorium development is cell cycle-regulated in *M. oryzae* with checkpoints regulating initiation and maturation of the appressorium [3], [4]. Differentiation of the appressorium is accompanied by autophagy in the conidium leading to programmed cell death and mobilisation of the contents of the three-celled spore to the infection cell. Prevention of autophagy by deletion of any of the core genes associated with non-selective macroautophagy, renders the fungus non-pathogenic, demonstrating that re-cycling of the contents of the conidium is essential for the appressorium to function correctly [3], [5].

The enormous turgor generated by the *M. oryzae* appressorium is the result of glycerol accumulation, which acts as a compatible solute, causing influx of water into the cell to create hydrostatic pressure [6]. Efflux of glycerol is prevented by a layer of melanin in the appressorium cell wall and mutants unable to synthesize melanin cannot generate turgor and are consequently non-pathogenic. Previously, glycogen reserves and lipid bodies were shown to move from the conidium to the *M. oryzae* appressorium prior to turgor generation [7]–[9]. This process is controlled by the Pmk1 MAP kinase pathway, which regulates appressorium morphogenesis [10] and is likely to be linked to the onset of autophagy in the conidium [3]. Lipid and glycogen breakdown in the appressorium are controlled by the cAMP response pathway and *cpkA* mutants, which lack protein kinase A activity, show significant delays in lipid and glycogen breakdown [7]. The rapid changes in primary metabolism during appressorium maturation appear to be regulated in part by a trehalose-6-phosphate synthase (Tps)-mediated genetic switch, which responds to levels of glucose-6-phosphate (G6P) and the NADPH/NADP balance in cells [11]. The Tps-mediated gene switch interacts with three transcriptional inhibitors which regulate virulence-associated gene expression in response to prevailing metabolic conditions [11].

In this study, we investigated the role of glycogen metabolism in the function of the *M. oryzae* appressorium. We show that glycogen reserves in the spore are broken down rapidly during spore germination, in a process regulated by the cAMP response pathway. We demonstrate that the *GPH1* glycogen phosphorylase and *AGL1* amyloglucosidase genes, which encode enzymes required for cytosolic glycogen breakdown, are virulence factors involved in plant infection. Surprisingly, however, we also show that glycogen synthase, which is encoded by the *GSN1* gene in *M. oryzae*, is dispensable for pathogenicity. To explain this apparent paradox, we provide evidence that loss of amyloglucosidase or glycogen phosphorylase activity in *M. oryzae* leads to a reduction in the expression of *TPS1*, thereby affecting G6P/NADPH-dependent gene regulation [11]–[13]. Consistent with this idea, we show that deletion of *NMR3*, one of the transcriptional inhibitors associated with Tps1, partially restores virulence to a Δ*gph1Δagl1* mutant. Our results suggest that glycogen breakdown in the appressorium is a significant factor in regulating virulence-associated gene expression.

## Results

### Glycogen mobilisation during infection-related development of *Magnaporthe oryzae*

**Figure 1:**
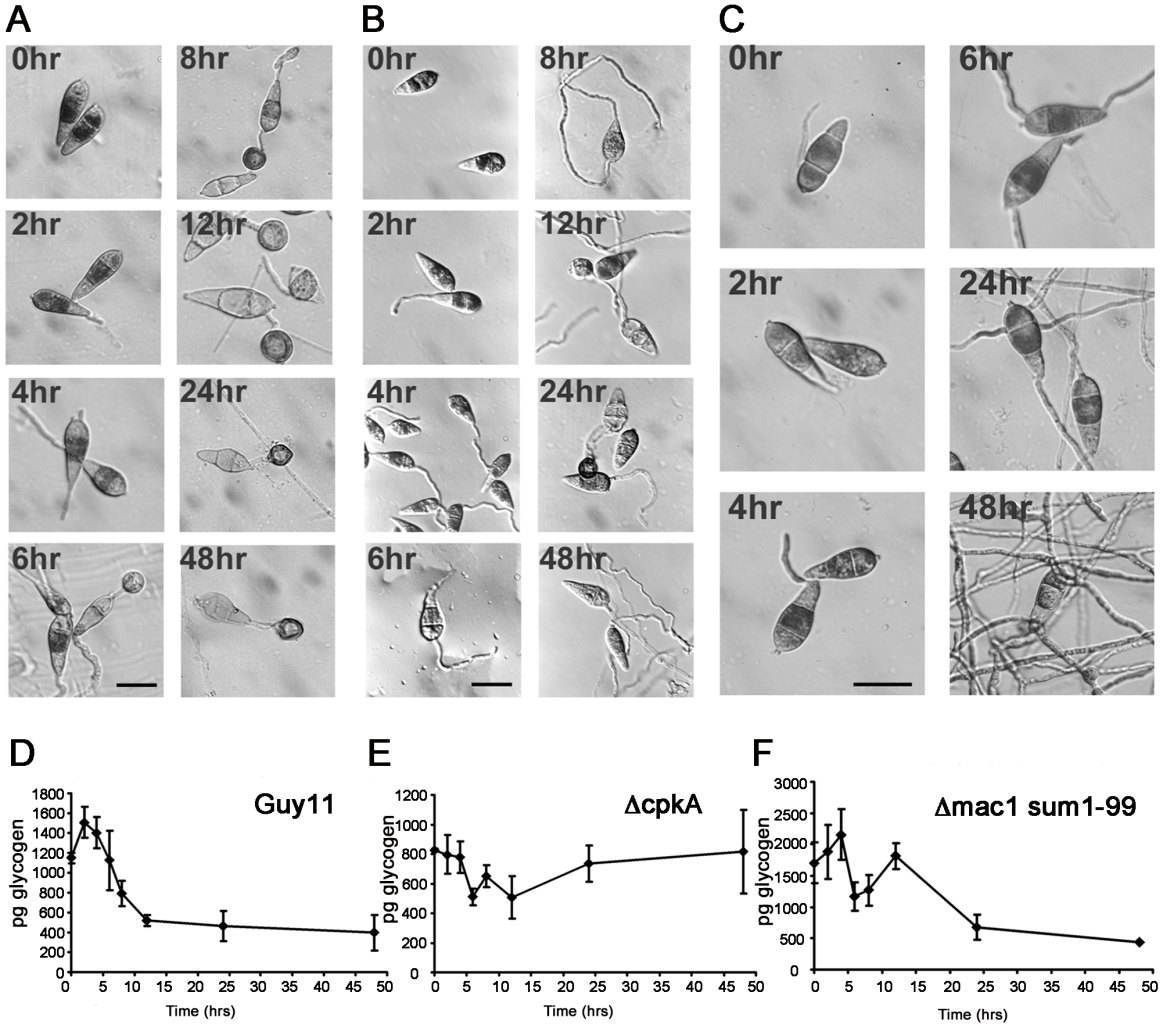
Cellular distribution and quantitative analysis of glycogen during appressorium morphogenesis in *M. oryzae*. Micrographs of glycogen mobilisation during appressorium development by *M. oryzae*. Conidia from strains of *M. oryzae* were germinated on hydrophobic plastic cover slips. Samples were removed at 0 h, 2 h, 4 h, 6 h, 8 h, 12 h, 24 h and 48 h and stained for the presence of glycogen with iodine solution. Yellowish-brown glycogen deposits became visible immediately using Hoffman modulation optics with a Nikon Optiphot-2 microscope. **A**. Wild type strain Guy 11 germinated in water. **B**. *ΔcpkA* mutant DF51. **C**. Wild type strain Guy 11 germinated in complete medium (CM) containing 2% yeast extract. Bar = 10 µm. **D–F** Graphs to show biochemical analysis of glycogen mobilisation during appressorium formation by *M. oryzae* Conidia were germinated in water on hydrophobic plastic cover slips and allowed to form appressoria. The amount of glycogen present in picograms was estimated by measuring the glucose released after incubation of the cellular extracts with glycogen degrading enzymes. **D**. Wild type strain Guy 11. **E**. *ΔcpkA* mutant DF51. **F**. *Δmac1 sum1-99* mutant DA-99. Each data point represents the mean of three independent replications of the experiment. Error bars show the standard deviation.

To investigate the role of glycogen mobilisation during appressorium formation, we first studied the cellular distribution of glycogen granules in a *M. oryzae* wild-type strain, Guy-11 and regulatory mutants affected in appressorium morphogenesis. In Guy-11, un-germinated conidia (0 h incubation) were glycogen-rich, indicated by a dark precipitate in each of the three conidial cells after incubation in iodine solution (Figure 1A), as previously described [7]. During germination and early appressorium formation (2–4 h), glycogen was degraded, with residual glycogen located only within the central cell of the conidium. After 6 h germination, glycogen appeared in the nascent appressorium, but was rapidly depleted during appressorium maturation, until at 24 h only the dark melanin ring around the appressorium and small number of glycogen grains were visible (Figure 1A, [7]).

In order to investigate the role of the cAMP pathway in glycogen mobilisation, we investigated a *ΔcpkA* mutant (Figure 1B). *CPKA* encodes the catalytic subunit of cAMP-dependent protein kinase A [14], [15]. Conidia were initially rich in glycogen, but glycogen degradation in *ΔcpkA* mutants was delayed with glycogen still present in conidia at 8 h. Glycogen was then deposited in misshapen *ΔcpkA* appressoria, but these cells did not fully develop and glycogen failed to be degraded (Figure 1B). We conclude that the cAMP pathway regulates efficient degradation of glycogen during germination and appressorium formation. Consistent with this idea, the cAMP bypass suppressor mutant *Δmac1 sum1-99* [16], exhibits accelerated glycogen breakdown in appressoria, as previously reported [7].

To determine whether glycogen mobilisation is associated with appressorium development, Guy-11 conidia were germinated under high nutrient conditions (2% yeast extract) which suppress formation of appressoria [16]. Limited degradation of glycogen deposits was observed and after 48 h glycogen granules could be seen within branched germ tubes (Figure 1C). Mobilisation of glycogen from the conidium is therefore associated with appressorium morphogenesis in nutrient-free conditions.

### Biochemical analysis of glycogen mobilisation in the developing appressorium

We next developed an assay to allow quantification of glycogen reserves and found that un-germinated Guy11 conidia contain around 1200 pg of glycogen (Figure 1D). We observed 1500 pg glycogen germling^−1^ after 2 h germination, during germ tube emergence, but between 2 and 12 h after germination, the amount of glycogen fell to 500 pg germling^−1^ and remained at this level during appressorium maturation, representing an average decrease of 988 pg with 95% family wise confidence interval (667, 1310), *p*<0.001. Over the entire 48 hour time course average glycogen levels dropped by 751 pg (95% CI 1073, 430 *p*<0.001). To compare these results with previous measurements of glycogen levels in budding yeast *Saccharomyces cerevisiae* [17], they must be converted to femto moles (fmol) per cell. The number of *M. oryzae* cells can only be easily defined at 0 h (3-celled conidium) and 48 h (one-celled appressorium). An un-germinated *M. oryzae* conidium therefore contains an average of 2363 fmol glycogen cell^−1^, while an appressorium contains 2450 fmol glycogen cell^−1^. The significant glycogen storage capacity of wild-type *M. oryzae* cells is emphasised when these values are compared to the 19.5 fmol glycogen measured in an *S. cerevisiae* yeast cell [17]. The *ΔcpkA* mutant contained lower levels of glycogen than Guy11 (Figure 1E). At 0 h we detected 800 pg glycogen germling^−1^. There were no significant changes in *ΔcpkA* glycogen levels when considered over the entire period of the time course (overall average decrease 13 pg [95% CI 354, 328 *p* = 1]). In the cAMP-independent PKA mutant strain *Δmac1 sum1-99* the glycogen assay revealed initially high levels of glycogen of 1700 pg germling^−1^ (Figure 1F). This continued to increase to a peak at 2100 pg germling^−1^ after 4 h (average increase from 0 h to 4 h of 450 pg [95% CI −265, 1164 *p* = 0.545]). Between 4 and 6 h after germination, at the time of appressorium formation, glycogen levels decreased rapidly by an average of 986 pg germling^−1^ (95% CI 1700, 272 *p*<0.001). Levels appeared to rise again until 12 h, peaking at the lower value of 1800 pg germling^−1^ (average increase 646 pg [95% CI −152, 1445 *p* = 0.2156]. Glycogen then decreased gradually by an average of 1340 pg over the remaining 36 hours (95% CI 2139, 541 *p*<0.001) to a final value of approximately 400 pg germling^−1^ by 48 h. Over the entire time course glycogen levels in the *Δmac1 sum1-99* mutant decreased by an average 1230 pg (95% CI 1944, 516 *p*<0.001). We conclude that the cAMP response pathway regulates glycogen mobilisation during appressorium development in *M. oryzae*.

### Identification and characterisation of *M. oryzae AGL1* and *GPH1*

To determine whether glycogen mobilisation is necessary for pathogenicity of *M. oryzae* we identified the major enzymes associated with its breakdown. Glycogen is comprised of chains of glucose linked by α-1,4-glycosidic bonds and at approximately every tenth residue, a branch point is formed by an α-1,6-glycosidic bond. Amyloglucosidase (glycogen de-branching enzyme) removes α-1,6-glycosidic bonds from the highly branched polymer, to release glucose. A putative amyloglucosidase-encoding gene, *AGL1* (MGG_00063.6), was identified in the genome sequence of *M. oryzae* [18]. *AGL1* has a 4749 bp open reading frame interrupted by two introns of 134 bp and 91 bp and encodes a putative 1583 aa protein. Alignment of the potential *M. oryzae* Agl1 protein with amyloglucosidases from other organisms (Figure S1) revealed 72% identity to a hypothetical protein from *Neurospora crassa* and 47% identity to the glycogen de-branching enzyme Gdb1 of *S. cerevisiae* [19]. Further analysis of the protein sequence revealed four conserved stretches of amino acids present in the α/β barrel domain of all members of the α-amylase superfamily (data not shown). Breakdown of glycogen also requires the action of glycogen phosphorylase. This enzyme complements the α-1,6-glycosidic activity of amyloglucosidase, by attacking the exoglycosidic α-1,4-glycogen bonds of glycogen, cleaving and phosphorylating sequentially from non-reducing ends of the polymer. The *M. oryzae GPH1* (MGG_01819.6) gene was identified and revealed a 2664 bp ORF encoding a putative 888 aa protein. Two in-frame ATG start sites were present in the sequence, the most 5′ of these has the start site indicator nucleotide, A, at −3 bp [20] which is likely to be the translation initiation site for *GPH1* [21]. The protein showed 78% identity to a hypothetical protein in *N. crassa* and 74% identity to a putative glycogen phosphorylase from *Aspergillus fumigatus* (Figure S2). In *S. cerevisiae* a phosphorylation site is present in the N-terminal region of the protein [22]. The *M. oryzae* protein shows a high degree of similarity to this sequence and the threonine residue, at which phosphate is bound in the yeast glycogen phosphorylase, is conserved. In addition, all glycogen phosphorylases possess a binding site for pyridoxal-5′-phosphate, a co-factor involved in phosphorylation. The aldehyde group of this pyridoxine derivative forms a Schiff base with a specific lysine side chain of glycogen phosphorylase [23]. The amino acid sequence surrounding this site has been identified in yeast glycogen phosphorylase [22] and is highly conserved in *M. oryzae GPH1* with 17 out of 18 residues identical between the two organisms (data not shown). The 3021 bp genomic DNA sequence of *M. oryzae GPH1* was also identified from the published genome sequence [18]. Comparison of this genomic sequence with the *GPH1* cDNA sequence identified the presence of four introns.

### Targeted gene replacement of *M. oryzae AGL1* and *GPH1*

To examine the role of *AGL1* and *GPH1* in pathogenicity of *M. oryzae*, we performed targeted gene replacement (Figure S3A). A 2.8 kb *Hin*dIII fragment (Figure S3B), containing the majority of the 5′ region of *AGL1*, was removed and replaced with the hygromycin phosphotransferase cassette [24] conferring resistance to hygromycin B. The resulting construct (Figure S3B) was introduced into Guy-11 and transformants verified by DNA gel blot (Figure S3C). To delete *GPH1*, a 2.2 kb portion of the 3 kb coding sequence was subsequently replaced with the 1.4 kb hygromycin phosphotransferase resistance cassette (Figure S3D). The resulting cassette was introduced into Guy-11 and transformants selected (Figure S3E).

**Figure 2:**
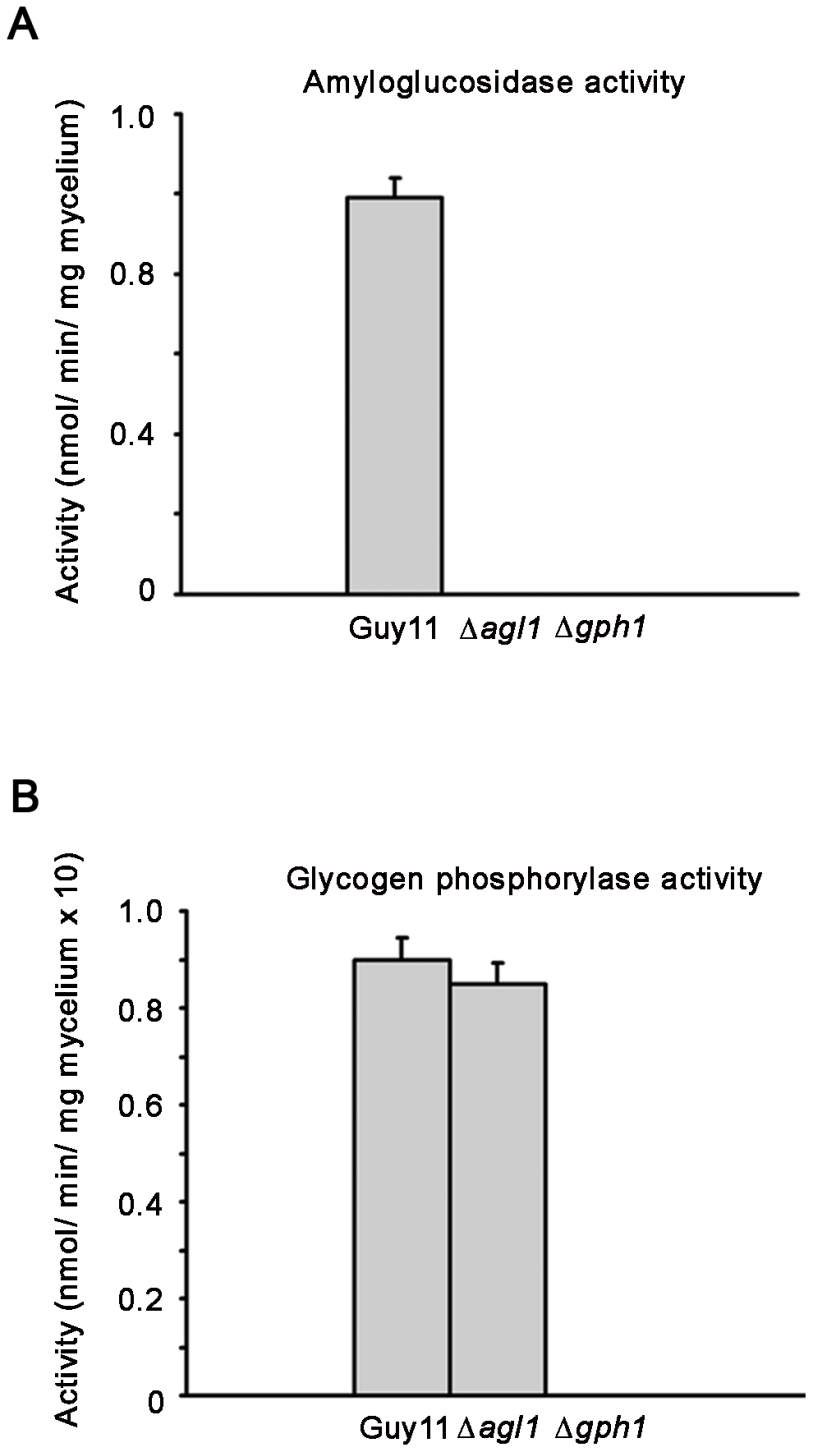
Bar charts to show amyloglucosidase and glycogen phosphorylase activity in *M. oryzae* glycogen metabolic mutants. For details of enzymatic assays see Materials and Methods. **A**. Amyloglucosidase activity was not detected in Δ*agl1* mutants or, as a consequence of the assay used, in Δ*gph1* strains. **B**. glycogen phosphorylase activity was absent in a Δ*gph1* mutant.

Deletion of each gene was confirmed by measurement of Agl1 and Gph1 enzyme activities (Figure 2A and B). Amyloglucosidase activity was not detectable in the Δ*agl1* strain and glycogen phosphorylase activity was not detected in Δ*gph1* mutants. We also observed, however, that amyloglucosidase activity could not be detected in Δ*gph1* deletion strains. Amyloglucosidase cleaves glycogen branch points only when they have been made accessible by the action of glycogen phosphorylase and was therefore not measurable in this assay.

To generate a Δ*agl1*Δ*gph1* double mutant, we carried out targeted deletion of *AGL1* in a Δ*gph1* mutant using the split marker gene deletion method [25]. Gene deletion constructs were transformed into Δ*gph1* using an allele of acetolactate synthase gene conferring resistance to sulfonylurea. The resulting transformants were selected and analysed by DNA gel blots (Figure S4). Additionally, RT-PCR was carried out to confirm successful gene deletion. In Guy11 and Δ*gph1*, a 789 bp amplicon corresponding to the Agl1 transcript was amplified but this was not detected in Δ*agl1* mutants or the Δ*agl1*Δ*gph1* double mutant (Figure S4D).

### Δ*agl1* and Δ*gph1* mutants show altered glycogen metabolism

**Figure 3:**
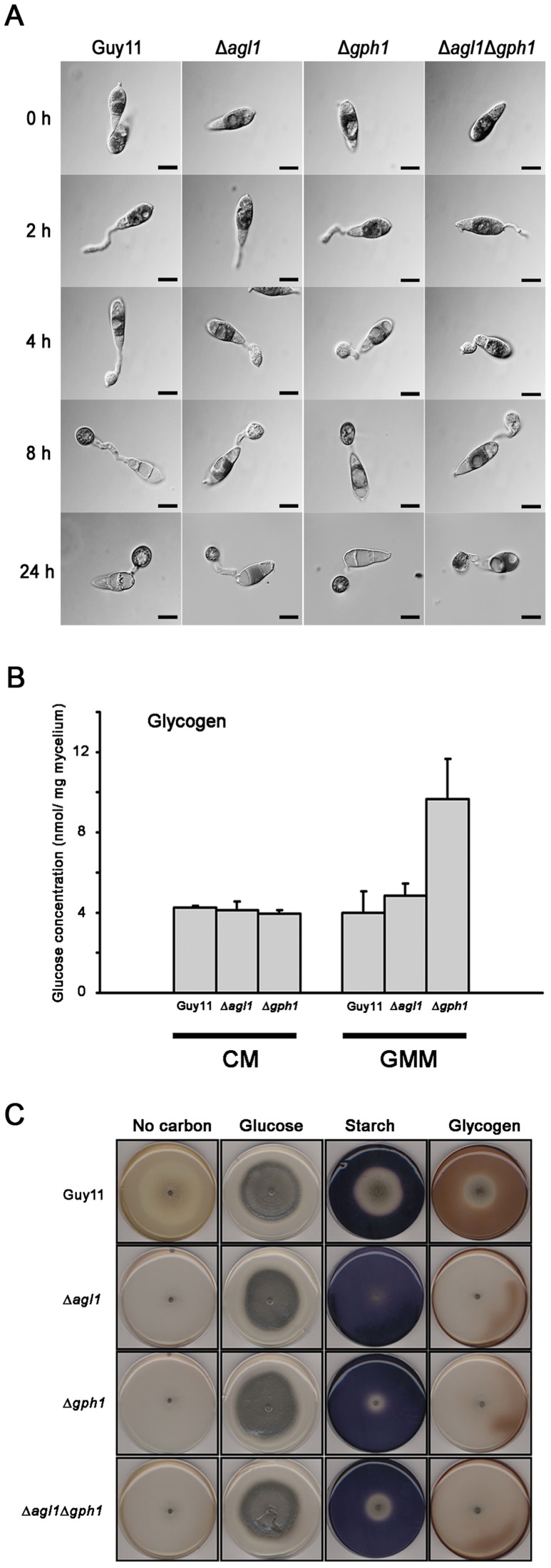
Cellular distribution and quantification of glycogen during appressorium morphogenesis in Δ*agl1* and Δ*gph1* mutants of *M. oryzae*. **A**. Conidia were incubated on hydrophobic glass coverslips to allow appressoria to develop before iodine solution staining. The Δ*agl1* and Δ*agl1*Δ*gph1* mutants were impaired in mobilisation of glycogen from the conidium to appressorium and in subsequent degradation in the appressorium, whereas the Δ*gph1* mutant was comparable to Guy11. Scale Bar = 10 µm. **B**. Glycogen quantification in mycelium extracts of the wild type, and glycogen metabolic mutants of *M. oryzae*. Strains were grown either in liquid CM for 48 h or in CM followed by growth in GMM for 16 h. Cultures were harvested, washed with 0.2% CaCl_2_ and freeze dried. The amount of glycogen present in nmolmg^−1^ mycelium was estimated by measuring the glucose released after incubation of the cellular extracts with glycogen degrading enzymes. Values shown with different letters (a, b,) are statistically significant at p<0.01. **C**. Growth tests to show utilization of starch and glycogen as sole carbon sources. Minimal medium containing glucose, starch or glycogen were inoculated with mycelial plug of *M. oryzae* and incubated for 12 days. Starch and glycogen plates were flooded with iodine to visualize the difference in substrate utilization. Iodine stains starch blue and glycogen brown. Glycogen mutants were impaired in their ability to utilize both starch and glycogen as sole carbon source.

To investigate whether cellular distribution of glycogen is affected in mutants carrying targeted deletions of *AGL1* and *GPH1*, we analysed glycogen mobilisation during appressorium formation in *Δagl1*, *Δgph1* and Δ*agl1*Δ*gph1* mutants (Figure 3). Mobilisation of glycogen was retarded in Δ*agl1* and Δ*agl1*Δ*gph1* mutants and glycogen was not depleted from conidia until 24 h compared with 8–12 h in Guy11. By contrast, Δ*gph1* mutants appeared to less compromised in glycogen mobilisation. These results suggest that amyloglucosidase is more important for efficient degradation of glycogen during infection-related development than glycogen phosphorylase. This may reflect the ability of a recently described glucoamylase to perform the α-1,4-glycosidic activity of glycogen phosphorylase and thus potentially compensate for loss of this activity [25]. Glycogen phosphorylase is important for mycelial glycogen breakdown, however, as glycogen accumulated in Δ*gph1* strains during growth on minimal medium (Figure 3B), but not in Δ*agl1* strains.

To determine whether Δ*agl1*, Δ*gph1* and Δ*agl1*Δ*gph1* mutants are able to utilize external glycogen as sole carbon source, we carried out growth tests on a variety of carbon sources. Both Δ*agl1* and Δ*gph1* mutants, together with the Δ*agl1*Δ*gph1* double mutant, were able to utilize glucose, but were compromised in growth on glycogen and starch (Figure 3C). Re-introduction of *AGL1* and *GPH1* into corresponding null mutants complemented the mutant phenotype (Figure S5). We conclude that Δ*agl1* and Δ*gph1* mutants are impaired in their ability to utilise both starch and glycogen as sole carbon source.

### Agl1 and Gph1 localise to the cytoplasm of conidia, appressoria and invasive hyphae

**Figure 4:**
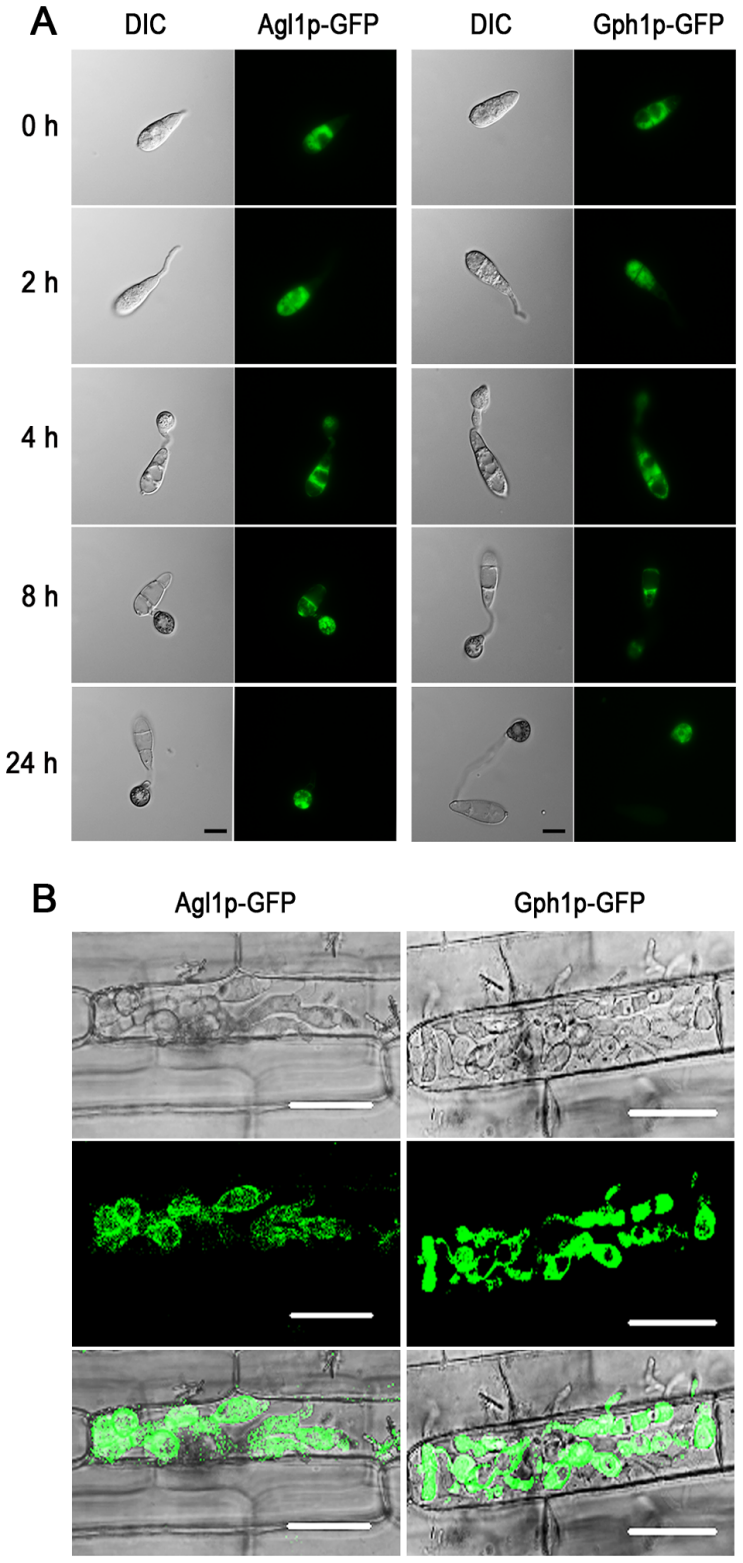
Localization of Agl1-GFP and Gph1-GFP during different stages of appressorium morphogenesis and *in planta* growth by *M. oryzae*. **A**. Conidia were allowed to form appressoria and localization of Agl1-GFP and Gph1-GFP observed during 24 h using an Olympus IX-81 inverted epifluorescence microscope fitted with HQ^2^ camera. Fusion proteins were localized to cytoplasm in conidium, germ tube and appressorium. Scale bar = 10 µm. **B**. Conidia were harvested from Agl1-GFP and Gph1-GFP strains and incubated onto rice leaf sheath in a moist chamber. A Zeiss LSM510 Meta confocal laser scanning microscope system was used to observe expression of Agl1-GFP and Gph1-GFP in rice epidermal tissue 36 h post incubation. Agl1-GFP and Gph1-GFP were expressed cytoplasmically in invasive hyphae. Scale bar = 20 µm.

To determine the sub-cellular location of Agl1p and Gph1p during appressorium formation, *AGL1:GFP* and *GPH1:GFP* gene fusions were expressed in Guy11. Agl1-GFP and Gph1-GFP fusions localized throughout the cytoplasm in conidia of Guy11 and within germ tubes and nascent appressoria by 2 h and 4 h, respectively (Figure 4A). After 8 h, the fluorescent signal was observed predominantly in the developing appressorium and by 24 h, Agl1-GFP and Gph1-GFP were found exclusively in the cytoplasm of the appressorium. Interestingly, Agl1-Gfp and Gph1-Gfp were also highly expressed during plant infection after 36 h and localized to the cytoplasm of the intracellular invasive fungal hyphae (Figure 4B).

### *M. oryzae* Δ*agl1*, Δ*gph1*, Δ*agl1*Δ*gph1* mutants are impaired in their ability to cause rice blast disease

**Figure 5:**
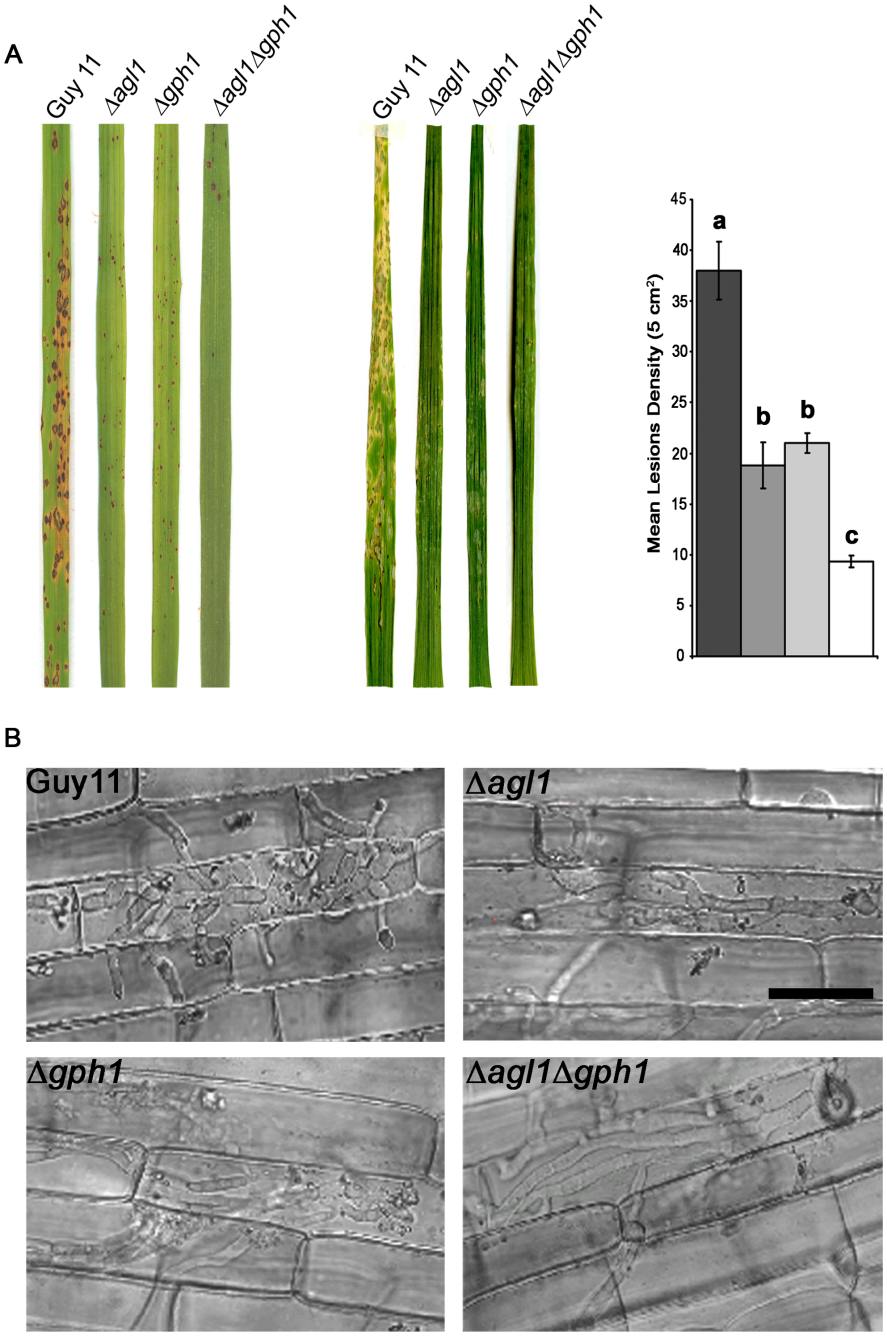
Infection and penetration assays of Δ*agl1*, Δ*gph1* and Δ*agl1Δgph1* mutants of *M. oryzae*. **A**. Fifteen-day-old seedlings of rice cultivar CO-39 or 8-day-old seedlings of barley Golden Promise were sprayed with a conidial suspension of 5×10^−4^ spores ml^−1^ and incubated at 24°C, with high light and 90% humidity for 5 days. Leaves shown represent typical symptoms associated with each strain. The experiment was repeated six times with similar results. Bar charts show lesion densities scored from infected rice leaves that were selected at random. A one-way ANOVA analysis showed that the mutant strains were significantly deficient in their ability to cause rice blast disease. Values shown with different letters (a, b, c) are statistically significant at p<0.01. **B**. To investigate host tissue invasion, conidia were incubated on rice leaf sheath for 36 h and observed by laser confocal microscopy. The wild type strain Guy11 showed growth with invasive bulbous and branched hyphae. Glycogen metabolic mutants produced less bulbous and branched invasive hyphae inside rice cells. Scale bar = 20 µm.

To determine the role of *AGL1* and *GPH1* in fungal pathogenicity, we inoculated the susceptible rice cultivar, CO-39, and barley cultivar, Golden Promise, with uniform spore suspensions of each mutant and Guy-11 (Figure 5A). The Δ*agl1* and Δ*gph1* mutants showed lesion numbers reduced by 50.44±6% (*P*<0.0001) and 44.7±2.4% (*P*<0.001), respectively, whereas the Δ*agl1*Δ*gph1* strain was reduced in lesion number by 75.44±1.4% (*P*<0.0001) (Figure 5A). The mutants showed no defects in conidial germination and appressorium formation under nutrient-free conditions and the frequency of appressorium formation was also unaffected (data not shown). It has previously been proposed that glycogen breakdown may be significant in appressorium turgor generation [6], [7], [26]. We therefore measured appressorium turgor in the Δ*agl1* and Δ*gph1* mutant strains using an incipient cytorrhysis assay [6]. Despite differences in glycogen mobilisation in the mutant strains (Figure 3), we found no significant differences in turgor between appressoria of the mutant strains and Guy-11 (not shown). We then used an onion epidermis assay [27] to determine whether the observed reduction in virulence could be a consequence of impaired appressorium function. However, no significant differences in epidermal penetration were observed between Guy-11 and the *Δagl1*, *Δgph1* and *Δagl1Δgph1* mutant strains which elaborated penetration pegs normally (not shown). Therefore, the reduction in lesion formation seen for Δ*agl1*, Δ*gph1* and *Δagl1Δgph1* is not a consequence of reduced turgor pressure or impaired appressorium function. To investigate tissue colonisation by each mutant, a rice leaf sheath assay was performed [28]. We found that Δ*agl1*, Δ*gph1* and Δ*agl1*Δ*gph1* mutants were defective in their ability to grow invasively inside rice cells. Glycogen metabolic mutants formed unusual invasive hyphae that were less bulbous and branched than normal (Figure 5B). We conclude that Δ*agl1*, Δ*gph1* and Δ*agl1*Δ*gph1* mutants are able to penetrate rice cells normally but cannot proliferate within rice tissue effectively, leading to reduced disease symptoms.

### Identification of a *M. oryzae* glycogen synthase encoding gene

Given the significance of glycogen mobilisation to virulence, we set out to make a strain of *M. oryzae* that was depleted in glycogen reserves. A putative glycogen synthase-encoding gene was identified from the *M. oryzae* genome database (http://www.broadinstitute.org/annotation/fungi/magnaporthe/) using the BLASTX algorithm [29] based on its predicted amino acid homology to yeast and mammalian glycogen synthases. One sequence, MGG_07289.6 aligned with yeast and mammalian glycogen synthases [30], [31]. The predicted glycogen synthase-encoding gene, which we named *GSN1*, is present as a single copy gene in the *M. oryzae* genome and predicted to contain five exons, interrupted by four introns [29]. The predicted gene encodes a 708 amino acid protein and showed high sequence similarity to Gsy1p and Gsy2p of yeast [30] and Gys1p and Gys2p of the human muscle and liver specific forms of glycogen synthase [31]. Figure S6 shows the result of a ClustalW alignment [32]. *S. cerevisiae* Gsy1 and Gsy2 showed 63% and 64% identity to *M. oryzae GSN1*, respectively whereas *Homo sapiens* Gys1 and Gys2 showed 60% and 55% identity, respectively. Phosphorylation sites putatively involved in post-translational regulation of Gsy2p were conserved in *M. oryzae* Gsn1p. In Gsy2p, three phosphorylation sites are present at S650, S654 and T667 in the C-terminus [33] whereas in Gsn1p the predicted residues are present at S632, S636 and T645 positions at the C-terminus, as shown in Supplemental Figure S5.

### Glycogen synthesis is dispensable for pathogenicity of *M. oryzae*

**Figure 6:**
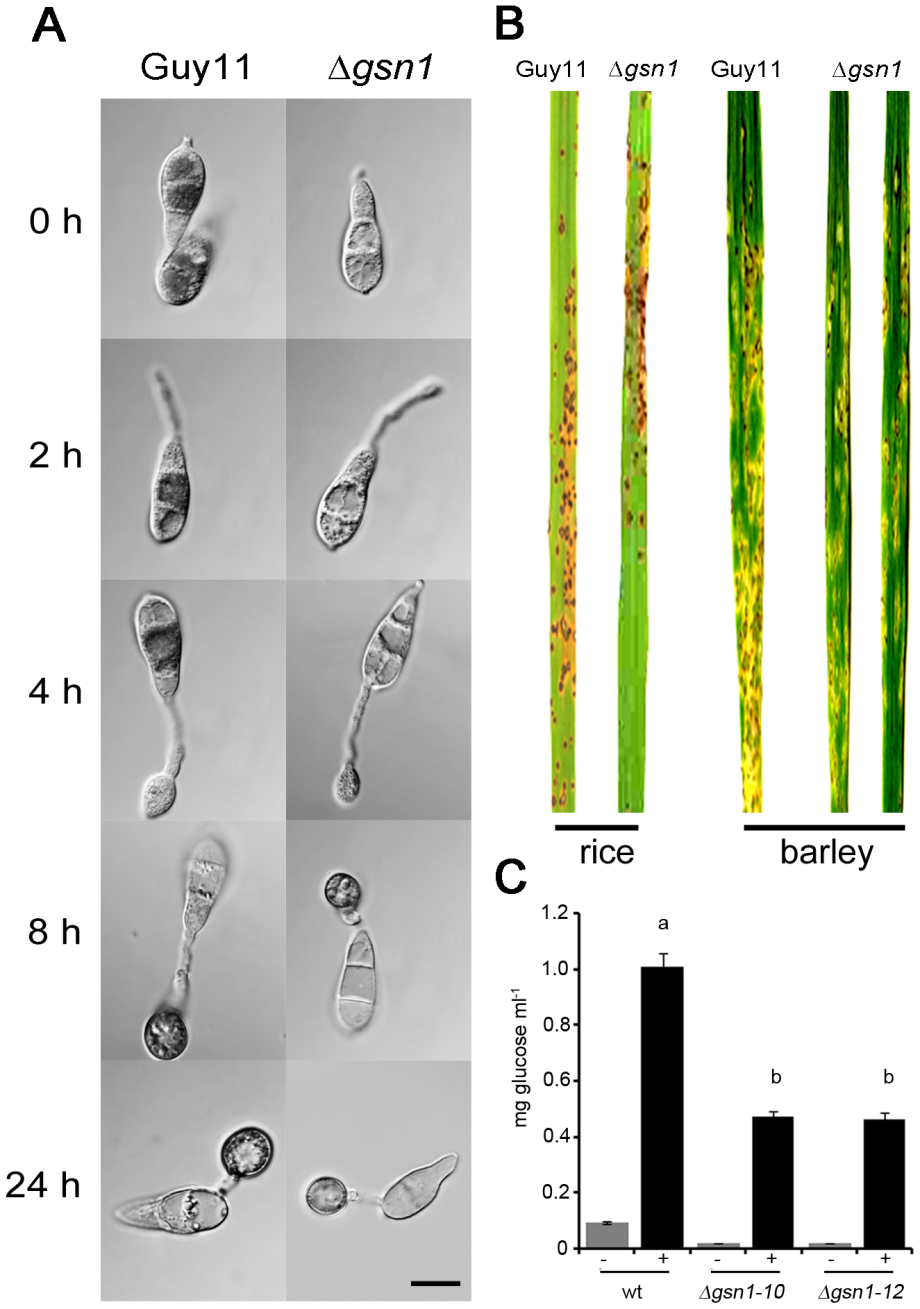
Cellular distribution of glycogen during appressorium morphogenesis in the glycogen synthase mutant Δ*gsn1* of *M. oryzae*. **A**. Conidia were allowed to form appressoria and then iodine solution staining performed. Glycogen deposits were absent in conidia and throughout appressorium development. Scale Bar = 10 µm. **B**. Infection assays of Δ*gsn1* mutants. Spores were harvested from 12-day-old cultures of Guy11 and the Δ*gsn1* mutant and spray inoculated at a concentration of 5×10^4^ conidia ml^−1^ on 3-week old rice seedlings and 8-day old barley seedlings. The plants were inoculated at 24°C with high light and 90% humidity for five days. Leaves were harvested and scored for rice blast symptoms. No obvious difference was observed between the pathogenicity of the wild type strain Guy11 and the glycogen synthase mutant Δ*gsn1*. The experiment was repeated three times with identical results and no statistically significant differences were observed in lesion density or severity (not shown). C. Glycogen quantification in conidia of the wild type and glycogen synthase mutants of *M. oryzae*. Conidia were harvested from 12-day-old plates of wild type and two Δ*gsn1* deletion mutants, and diluted to a concentration of 1×10^6^ ml^−1^. The amount of glycogen present in mg ml^−1^ conidia was estimated by measuring the glucose released after incubation of the cellular extracts with glycogen degrading enzymes (+). The amount of glucose estimated without the addition of glycogen degrading enzymes is also shown (−). Values with different letters (a, b,) are statistically significant at p<0.01.

To generate a Δ*gsn1* null mutant of *M. oryzae*, targeted gene replacement of *GSN1* was carried out using the split marker gene deletion method (Figure S6A) [25]. Gene deletion constructs were transformed into Guy11 and putative transformants screened based on their resistance to hygromycin B (Figure S7). In Δ*gsn1* mutants, glycogen deposits could not be clearly observed (Figure 6A), in contrast to Guy11 (Figure 1). To investigate this further, we measured glycogen using an enzymatic assay and observed a significant reduction (*P*<0.01) in glycogen within cells of the Δ*gsn1* mutant (Figure 6C). We conclude that glycogen synthesis is severely compromised in both conidia and appressoria in the absence of the *GSN1* glycogen synthase gene.

To investigate the role of *GSN1* in disease progression of rice blast disease, seedlings of rice CO-39 and barley cv. Golden Promise were each inoculated with the glycogen synthase mutant Δ*gsn1* and the wild type strain Guy11. The Δ*gsn1* mutant was able to cause rice blast disease symptoms, which were identical to the wild type strain from which it may be inferred that this gene is dispensable for rice blast infection in both rice and barley (Figure 6B). We conclude that the synthesis of glycogen as a storage product in spores in *M. oryzae* is not essential for pathogenicity.

### Δ*agl1*, Δ*gph1*, and Δ*agl1*Δ*gph1* mutants are affected in trehalose synthesis

**Figure 7:**
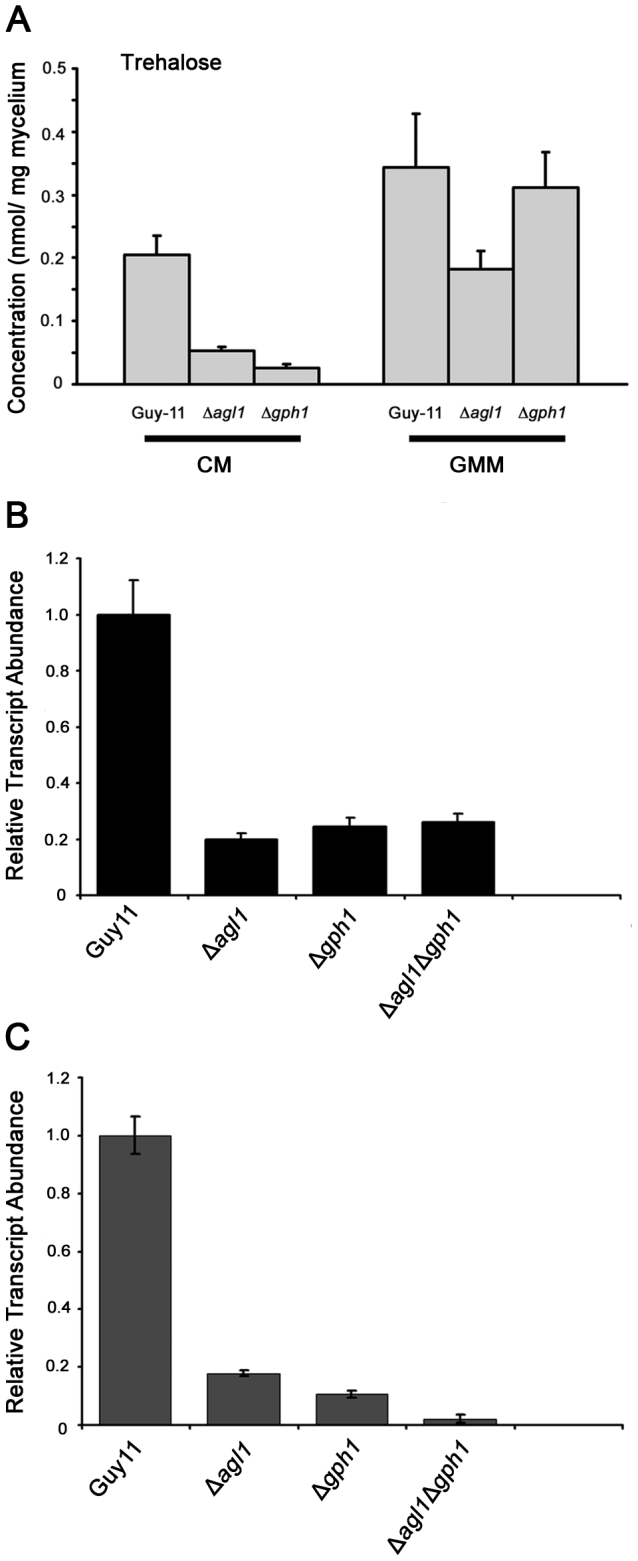
Analysis of trehalose synthesis in glycogen metabolic mutants of *M. oryzae*. **A**. Trehalose quantification in mycelial extracts. Strains were grown either in liquid CM for 48 h or in CM followed by growth in GMM for 16 h. Cultures were harvested, washed with 0.2% CaCl_2_ and freeze dried. Mycelial extracts were made with methylene dichloride and methanol and assayed for trehalose. Trehalose levels were significantly reduced in glycogen metabolism mutants when compared to Guy11. The experiment was repeated three times with similar results. Values shown with different letters (a, b, c, d) are statistically significant at p<0.01. **B**. Gene expression analysis of *TPS1* and **C**. *TPS3* in mycelium by comparative quantitative real-time RT-PCR. Transcript levels of *TPS1*, *TPS3* were down regulated in glycogen metabolic mutants. The experiment was repeated twice, each with three technical replicates, with similar results. A one-way ANOVA analysis showed that the mutant strains were significantly defective in trehalose biosynthetic gene expression. Transcript abundance is shown relative to expression of the *M. oryzae β*-tubulin gene (MGG_00604). Values shown with different letters (a, b, c, d) are statistically significant at p<0.01.

We were intrigued to find that impairing glycogen synthesis had no effect on virulence of *M. oryzae*, given that both Δ*agl1* and Δ*gph1* mutants showed such pronounced defects in their ability to cause rice blast disease. We therefore set out to identify whether other biochemical differences were apparent in either of the glycogen metabolic mutants. Interestingly, we observed that Δ*agl1* and Δ*gph1* mutants both showed reduced trehalose levels during vegetative growth on CM (Figure 7A). This suggests that in *M. oryzae*, the precursors of trehalose, at least during mycelium growth, may be derived by glycogen degradation, or are affected by loss of these enzyme activities. We therefore analyzed expression of *TPS1* and *TPS3* in Guy11, Δ*agl1*, Δ*gph1* and Δ*agl1*Δ*gph1* mutants. *TPS1* encodes trehalose-6-phosphate (T6P) synthase and *TPS3* encodes a subunit of the T6P synthase/trehalose phosphatase complex [34]. Expression of *TPS1* was reduced 5-fold in Δ*agl1*, Δ*gph1* and Δ*agl1*Δ*gph1* mutants compared to Guy11 (Figure 7B), whereas expression of *TPS3* was reduced 6-fold in Δ*agl1*, 9-fold in Δ*gph1* and 50-fold in the Δ*agl1*Δ*gph1* mutant (Figure 7C). We also investigated expression of the *RSY1* gene involved in the biosynthesis pathway of melanin [35], because *TPS1* is known to regulate the expression of several virulence-associated genes, including melanin biosynthetic genes via the *TPS1/NMR* genetic switch [11]. Expression of *RSY1* was also down-regulated in the independent single and the double mutants (not shown).

### Deletion of *NMR3* partially remediates Δ*agl1*Δ*gph1* double mutant phenotype

It is known that *M. oryzae* Tps1 acts as a G6P-sensing protein, directly binding to G6P and NADPH to control expression of a set of virulence-associated genes expressed during plant infection [11]. We hypothesized that if glycogen metabolic genes (*AGL1* and *GPH1*) regulate expression of *TPS1* during infection, then deletion of one of the transcriptional inhibitor genes that have been shown to interact with Tps1p might partially complement phenotypic defects associated with a Δ*agl1*Δ*gph1* double mutant [11]. Deletion of *NMR1*, *NMR2* or *NMR3*, for instance, has been shown to be sufficient to restore pathogenicity to a Δ*tps1* mutant [11]. We therefore carried out targeted deletion of the *NMR3* gene in a Δ*agl1*Δ*gph1* double mutant, because Δ*tps1*Δ*nmr3* double mutants showed maximum restoration of pathogenicity [11]. We employed a split marker strategy to target the *NMR3* locus for gene deletion, as shown in Figure S8A [25]. Following transformation, *M. oryzae* transformants were initially screened for resistance to bialaphos [36] and the deletion confirmed by DNA gel blot (Figure S8B).

**Figure 8:**
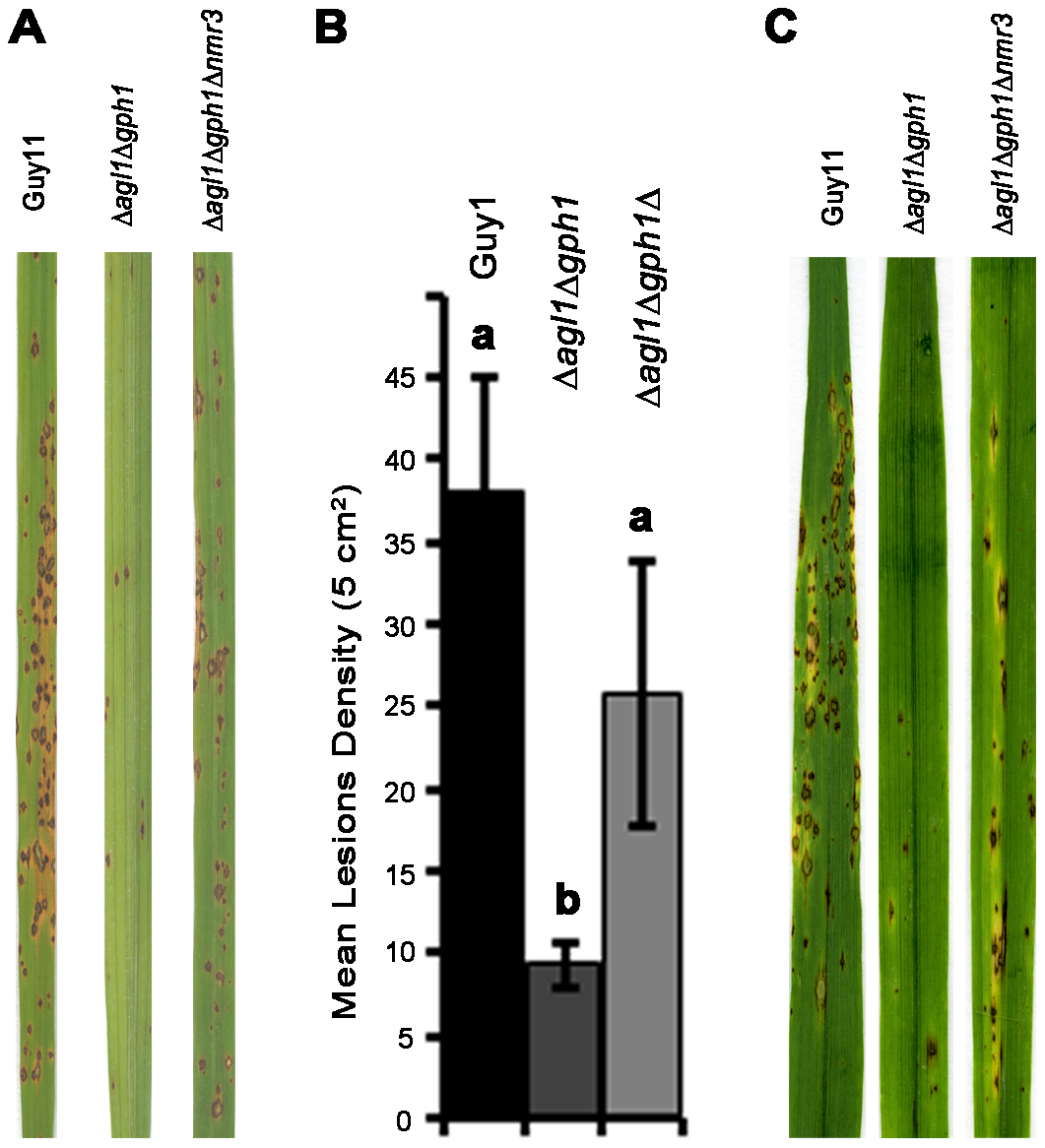
Rice blast assays of Δ*agl1*Δ*gph1* and the Δ*agl1*Δ*gph1*Δ*nmr3* mutant of *M. oryzae*. **A**. 3-week old rice seedlings of cultivar CO-39 were sprayed with a spore suspension of 5×10^4^ spores ml^−1^ and incubated for 5 days at 24°C. **B**. Lesions were scored from randomly selected infected leaves. Leaves shown represent typical symptoms associated with each tested strain. Experiments were repeated six times with identical results. The Δ*agl1*Δ*gph1*Δ*nmr3* was partially restored in its ability to cause blast disease. Values shown with different letters (a, b) are statistically significant at p<0.01. C 8-day-old seedlings of barley Golden Promise were sprayed with a conidial suspension of 5×10^−4^ spores ml^−1^ and incubated at 24°C, with high light and 90% humidity for 5 days. Leaves shown represent typical symptoms associated with each strain. The experiment was repeated three times with similar results.

To test the virulence of Δ*agl1*Δ*gph1*Δ*nmr3* on the susceptible rice cultivar CO-39, a pathogenicity assay was carried out using Guy11, an Δ*agl1*Δ*gph1* mutant and the Δ*agl1*Δ*gph1*Δ*nmr3* mutant. We found that *NMR3* gene deletion restored the ability of the Δ*agl1*Δ*gph1* mutant to cause blast disease as shown in Figure 8. No significant difference in lesion number was observed between Guy11 and Δ*agl1*Δ*gph1*Δ*nmr3* in rice infection (*P*<0.05), or barley infections (Fig. 8). We conclude that *AGL1* and *GPH1* may influence the operation of Tps1-mediated gene regulatory mechanism that is necessary for rice blast disease.

## Discussion

In this study, we set out to investigate the role of glycogen metabolism in establishment of rice blast disease by *M. oryzae*. Previous reports have established that glycogen mobilization occurs during appressorium development and may depend on cAMP-dependent protein kinase A and the Pmk1 MAP kinase signalling pathways [7]. Glycogen reserves in the appressorium have also been proposed as a potential substrate for glycerol production in the appressorium, facilitating generation of turgor to rupture the plant cuticle [6], [7], [26]. Glycogen reserves in appressoria are, for instance, clearly utilized during appressorium maturation, prior to plant infection [26]. When considered together, the results presented in this study provide evidence that cytosolic glycogen degradation in *M. oryzae*, which requires glycogen phosphorylase and amyloglucosidase, plays an important role in the virulence of this fungus. This conclusion is based on the mutant phenotypes of Δ*agl1*, Δ*gph1* and Δ*agl1*Δ*gph1* double mutants, which are impaired in their ability to cause blast disease. However, it seems likely that the effect on virulence is pleiotropic, rather than specifically associated with utilization of glycogen reserves, because Δ*gsn1* mutants that are significantly reduced in glycogen reserves due to absence of glycogen synthase activity, are unaffected in virulence. This result suggests that glycogen reserves in the conidium and appressorium are dispensable for appressorium turgor generation and plant infection. Data presented here also suggests that loss of *GPH1* and *AGL1* reduces expression of *TPS1* which is known to integrate control of carbon and nitrogen metabolism in *M. oryzae* in response to G6P and NADPH/NADP levels [11].

At the outset of this study, we were particularly interested in understanding how glycogen mobilization occurred during appressorium development by *M. oryzae*. We therefore initially carried out both cytological and biochemical analysis to study the movement and breakdown of glycogen stores. The results of these experiments were consistent in showing that glycogen is rapidly degraded during conidial germination and that the process is retarded in Δ*cpkA* mutants lacking the catalytic sub-unit of protein kinase A. Conversely, the Δ*mac1 sum1-99* mutant, a bypass suppressor of an adenylate cyclase null mutant that carries a mutation in the cAMP-binding pocket of the regulatory sub-unit of protein kinase A [16], shows mis-regulation in glycogen storage in the spore. This is consistent with earlier suggestions, based solely on microscopy [7], that Δ*mac1 sum1-99* mutants are accelerated in glycogen and lipid breakdown. Taken together, these observations suggest that the cAMP response pathway is significant in regulating cytosolic glycogen metabolism.

To investigate the role of glycogen breakdown in plant infection by *M. oryzae*, we generated gene deletion mutants lacking amyloglucosidase and glycogen phosphorylase enzyme activities. Phenotypic analysis revealed that Δ*agl1* and Δ*agl1*Δ*gph1* double mutants were severely defective in degrading glycogen within the conidium and, after 24 h, glycogen rosettes remained visible in conidia of both mutant strains. Hyper-accumulation of glycogen has previously been observed in Δ*agl1* and Δ*gph1* mutants of *S. cerevisiae* [37], [38], demonstrating the significance of these enzymes to utilization of glycogen reserves. A previous report has shown that a vacuolar glucoamylase, Sga1, in *M. oryzae* may contribute to glycogen utilization during conidiogenesis [39]. Mutants lacking Sga1 activity showed reduced conidiation, consistent with a role for vacuolar, autophagic hydrolysis of glycogen during spore development. Interestingly, Δ*sga1* mutants did not, however, show any effect on appressorium development or pathogenicity [39], suggesting that *M. oryzae* may degrade glycogen through a vacuolar autophagic mechanism during spore development, but a largely cytosolic route during spore germination and appressorium development, even though a large burst of autophagy is associated with conidial germination, programmed cell death and appressorium maturation [3], [5]. Agl1-GFP and Gph1-GFP gene fusions, for instance, confirmed that both enzymes are localized in the cytoplasm within conidia, germ tubes and appressoria.

The phenotypes of Δ*agl1* and Δ*gph1* mutants are consistent with glycogen mobilization and utilization having a significant effect on the ability of *M. oryzae* to cause rice blast disease. However, deletion of *GSN1* which encodes glycogen synthase resulted in a significant reduction in glycogen levels within cells, but had no effect at all on pathogenicity of *M. oryzae*.. Lipid, glycogen and trehalose are the major nutrient reserves used by *M. oryzae* conidia for germination and appressorium differentiation (reviewed in [2]). Based on results presented here, it seems that glycogen utilization may not be as significant a factor in appressorium maturation as triacylglycerol hydrolysis [9]. Triacylglycerol lipase activity is, for instance, induced during appressorium development in *M. oryzae* [7].

The most significant conclusion we can make regarding the role of *AGL1* and *GPH1* in pathogenicity of *M. oryzae* is that the absence of these enzyme activities reduces expression of the *TPS1* gene. In *M. oryzae*, it is known that Tps1 activity is essential for rice blast disease and forms an NADPH-dependent genetic switch [11]. Mutants lacking Tps1 are able to produce appressoria, but they are non-functional and the fungus cannot colonize rice tissues [40]. The Δ*tps1* mutant does not produce trehalose or its intermediate trehalose-6-phosphate (T6P), but impairment of pathogenicity is not due simply to loss of trehalose biosynthesis. Mutations in the G6P-binding pockets of Tps1, for instance, rendered the fungus non-pathogenic, whereas mutations in the catalytic sites necessary for T6P synthesis did not affect its role in rice blast disease, suggesting that loss of G6P binding is associated with loss of pathogenicity [12]. Tps1 integrates carbon and nitrogen source utilization and Δ*tps1* mutants are unable to grow on nitrate due to effects on nitrate reductase (NR) activity [34]. NADPH is a co-factor for nitrate reductase and provides reducing power. Sufficient NADPH pools are necessary for the function of NR and are produced from the oxidative pentose phosphate pathway, via activation of G6P dehydrogenase (G6PDH). Both NADPH levels and G6PDH activity have been shown to be reduced in Δ*tps1* mutants, indicating that G6PDH expression is under the control of Tps1 [34]. Over-expression of G6PDH restores the pathogenicity defect of Δ*tps1* mutants and Tps1 has recently been shown to bind directly to NADPH, suggesting a wider regulatory role on carbon and nitrogen utilization [11]. Tps1 has also been shown to be a positive regulator of three GATA transcriptional factors in a process that is negatively regulated by three NADP-dependent Nmr inhibitor proteins. The GATA transcriptional factors positively regulate expression of virulence-associated gene expression in a manner modulated by the activity of the NMR inhibitor proteins and are, ultimately, dependent on cellular NADPH/NADP and G6P levels [11]. Strikingly, we observed that Δ*agl1* and Δ*gph1* mutants are reduced in trehalose accumulation. Moreover, the trehalose biosynthesis enzyme-encoding genes, *TPS1* and *TPS3*, are down-regulated in glycogen metabolic mutants. We therefore hypothesized that Agl1 and Gph1 might regulate expression of Tps1 and Tps3. Deletion of *NMR3* was sufficient to restore virulence to a Δ*agl1*Δ*gph1* double mutant, albeit with small lesions. This suggests that glycogen metabolism may contribute to control of the NADPH-dependent Tps1 genetic switch. This is interesting because *AGL1* and *GPH1* gene expression, and glycogen turnover, has been shown to be affected in Δ*tps1* strains [34], suggesting a negative-feedback mechanism might exist in wild type to regulate glycogen turnover in response to G6P sensing. When considered together, our study has therefore revealed fundamental new information regarding the role of glycogen metabolism in the rice blast fungus *Magnaporthe oryzae*. Results presented here suggest that glycogen metabolism exerts a wider regulatory role than was initially predicted but there is unlikely to be a direct requirement for glycogen degradation to fuel appressorium-mediated cuticle penetration. Instead the effect of Agl1p and Gph1p on the Tps1-dependent genetic switch suggests an important and unexpected regulatory role during plant infection.

## Materials and Methods

### Fungal strains and culture conditions

Isolates of *Magnaporthe oryzae* used in this study are stored in the laboratory of N. J. T. Targeted gene replacement mutants described are isogenic to the wild-type rice (*Oryza sativa*) pathogenic *Magnaporthe* strain Guy-11. Media composition and procedures for fungal culture and maintenance, nucleic acid extraction and DNA-mediated transformation were described previously [41].

### Analysis of intracellular glycogen

Glycogen within developing appressoria was visualised using an iodine stain, consisting of 60 mg KI and 10 mg I_2_ mL^−1^ in distilled water [42]. Freshly harvested conidia at a concentration of approximately 1×10^5^ mL^−1^ were incubated on hydrophobic plastic cover slips in either standard growth media (CM) with 2% yeast extract added or in distilled water for defined lengths of time. After a few seconds contact with the stain yellow/brown glycogen deposits could be observed microscopically. To measure the amount of glycogen present at stages of *M. oryzae* development a protocol was developed based on experiments performed in *S. cerevisiae* [17], [43]–[46]. Sporulating cultures of 12-day-old *M. oryzae* were flooded with sterile distilled water and scraped with a glass spreader. The resulting suspension was filtered through sterile miracloth (Calbiochem) and conidia recovered by centrifugation at 20°C (5,000 *g*, 10 minutes). After washing in distilled water the conidia were concentrated by centrifugation and diluted to approximately 1×10^6^ conidia mL^−1^. Samples were divided and incubated on hydrophobic plastic coverslips for time periods of 0, 2, 4, 6, 8, 24 or 48 hours before being transferred to microfuge tubes, heated to 100°C for 10 minutes to halt enzymatic action and cooled on ice. Conidia were then disrupted by 10 minutes of sonication, performed in 10-second bursts using a Vibracell sonicator (Sonics and Materials Inc., Danbury, U.S.A). Cell fragments were precipitated by centrifugation and 760 µL of the supernatant was removed to a fresh tube. To this, 100 µL 50 mM CaCl_2_ and 100 µl 0.5 M NaOAc pH 5.0 were added along with the following enzymes which convert glycogen to glucose: 29 µL amyloglucosidase (Roche, Germany), 10 µL α-amylase (Sigma). In a control experiment these enzymes were replaced by sterile distilled water. Reactions were incubated overnight at 57°C with constant rotation before centrifugation at 5,000 *g* for 3 minutes. For each time point the supernatant from twelve 50 µL aliquots was analysed with a Glucose oxidase assay kit (Sigma diagnostics) in which the final colour intensity is proportional to the glucose concentration. Sample absorbance values were measured at 450 nm using a Dynex Technologies MRX II plate reader (Jencons-PLS). The control with no added enzyme was used to measure the background level of glucose. The complete experiment was performed in biological triplicate on separate days and mean glycogen levels calculated for each time point. Glycogen levels were analysed by linear mixed effects models, for each mutant strain individually, with culture as a random effect and time (hrs) as a fixed effect. All pair-wise comparisons were then performed by Tukey HSD tests to maintain an overall test level of 5%. Means and differences in means are reported with 95% family wise confidence intervals and Tukey adjusted p-values. The *nlme* and *multcomp* packages in R v2.15 were used to perform statistical analyses.

### Identification and targeted gene replacement of *M. oryzae AGL1* and *GPH1*

Genomic DNA from *M. oryzae* was used for polymerase chain reaction (PCR) amplification with the primers AGL1 (5′-GCGTACATGGCCTGCTTTGAACGTA-3′) and AGL2 (5′-TGCTCTGTGTGGAGTAGGCACGAGA-3′), designed to conserved regions of an *AGL1* Expressed Sequence Tag (EST) sequence from the Clemson University Genetics Institute (CUGI) database (http://www.genome.clemson.edu/). The PCR was performed by applying 35 amplification cycles. After an initial denaturation for 5 minutes at 94°C the following amplification conditions were used: 45 seconds of denaturation at 94°C, 1 minute of annealing at 55°C, 1 minute of elongation at 72°C. This was followed by a final 10 minute extension at 72°C. The resulting 513 bp genomic DNA fragment was then used to screen a Guy-11 conidial cDNA library [15]. The true start codon and 5′ sequence of the identified *AGL1* cDNA were determined using a 5′ Rapid Amplification of cDNA ends (5′ RACE) strategy [47]. Screening of a λGEM-11 genomic library [41] with the *AGL1* cDNA clone identified a 6.5 kb *Kpn* I lambda genomic clone, named pLH1. The genomic sequence of this clone was obtained using a Genome Priming System (GPS) strategy. This Tn7 transposon-based system uses a transposase complex to randomly insert a transprimer into the dsDNA target [48], [49]. A population of products is produced each with the transprimer inserted at a different location, and unique priming sites on the end of each transprimer allow sequencing from both strands of the target DNA at the position of insertion. For gene replacement a 6.0 kb amplicon, consisting of the *AGL1* ORF with a 1.0 kb 5′ flank, was amplified from *M. oryzae* Guy-11 genomic DNA with primers AGLvectorF (5′-CCTGACGGATAATGGTGGGGTG-3′) and AGLvectorR (5′-CTTCGTCCGCAT CCATGTAGAG-3′), designed to the genome sequence of *M. oryzae* strain 70-15 (http://www.genome.wi.mit.edu/annotation/fungi/magnaporthe/). The PCR was performed with the following conditions: an initial 1 minute of denaturation at 95°C, then 30 cycles of 1 minute denaturation at 95°C, 1 minute annealing at 60°C and 6 minutes elongation at 72°C. This was followed by a final 10 minute extension at 72°C. The resulting amplicon was cloned into the pGEM-T vector (Promega) to create plasmid pLH3. pLH3 was then digested with *Hin*dIII to release a 2.8 kb fragment of the *AGL1* ORF and the linearised plasmid ligated to a 1.4 kb *Hin*dIII-linkered hygromycin B phosphotransferase gene cassette to create the gene deletion vector pLH3*H*.

The *M. oryzae GPH1* gene was identified in a similar manner to *AGL1*. Primers GPH1 (5′-CTTCCTCCAGTCAGTAGAGCG-3′) and GPH2 (5′-TTTGAGGAAGTCATAGCTACCG-3′) were designed from conserved regions of GPH-encoding genes using an EST sequence from the CUGI database showing a high degree of similarity to glycogen phosphorylase genes from *S. cerevisiae* [50], [51] and other organisms. A 319 bp fragment of *M. oryzae GPH1* was amplified by PCR amplification with the following conditions: an initial denaturation for 5 minutes at 94°C, then 35 amplification cycles comprising: 45 seconds of denaturing at 94°C, 1 minute of annealing at 54°C, 1 minute 30 seconds of elongation at 72°C. This was followed by a final 10 minute extension at 72°C. The resulting amplicon was gel-purified, cloned into the pBluescript vector (Stratagene) and used to screen a Guy-11 conidial cDNA library [15]. 5′ RACE [47] was used to determine the 5′ sequence and start codon of *GPH1* using a gene specific primer designed to the sequence of the longest cDNA clone. To construct the *GPH1* gene replacement vector a 5.1 kb amplicon, containing the *GPH1* ORF with approximately 1.0 kb flanks, was amplified from *M. oryzae* genomic DNA using the primers GPHnestedF (5′-GTTGCACTGAACCTCGAGTCTAGA-3′) and GPHnestedR (5′-TTCGCCAAGG ATGCTGGGCTCAAG-3′), designed using the genome sequence of the *M. oryzae* 70-15 strain. The standard PCR protocol was adapted to incorporate a *Taq*Plus® Long PCR system (Stratagene), designed for the amplification of long products. Samples were heated to 94°C for 5 minutes then the following conditions applied for 35 cycles: 30 seconds denaturation at 94°C, 30 seconds annealing at 55°C, 9 minutes elongation at 72°C. This was followed by a final 10 minute extension at 72°C. The resulting 5165 bp amplicon was gel-purified and cloned into the pGEM-T vector (Promega) to create plasmid pLH4. Digestion of pLH4 with *Bst*BI released a 2.2 kb fragment of the gene which was replaced with a 1.4 kb *Bst*BI-linkered *Hph* gene cassette to create the gene deletion vector pLH4*H*. Plasmids pLH3*H* and pLH4*H* were transformed into *M. oryzae* strain Guy-11. Single-copy insertion of plasmid DNA was confirmed for all transformants using DNA gel blot analysis.

Targeted gene replacement of *AGL1* in Δ*gph1* strain with sulfonylurea resistance marker, *ILV1*, to generate Δ*agl1*Δ*gph1* using split marker gene strategy (Catlett *et al.*, 2003). The replacement was achieved by replacing 1 kb of 5′ open reading frame of *AGL1* with 2.8 kb *ILV1* gene. In the first round of PCR, 1 kb of the flanking sequence from each side of the gene coding region (named LF and RF) was amplified using Agl1-50.1/Agl1-m13F and Agl1-30.1/Agl1-m13R primers, in combination, using genomic DNA of Guy11. Agl1-m13F and Agl1-m13R have additional 5′ sequences, corresponding to the M13F/M13R primers. This additional sequence facilitated overlapping fusion PCR to be carried out in the second round. In a parallel reaction, the selectable marker, *ILV1* was amplified in two halves using primers M13F/SUR-F and M13R/SUR-R from the sulfonylurea resistance cassette gene [36], and then cloned into pBluescript (Stratagene). These two products were named IL and LV from the *ILV1* gene. The first PCR was carried out under the following cycle conditions in an Applied Biosystems GeneAmp PCR System gradient cycler: an initial denaturation step at 94°C for 5 min, followed by 35 cycles of PCR cycling parameters, 94°C for 30 sec, 62°C or 30 sec and 72°C for 1 min, followed by final extension at 72°C for 10 min. Each 25 µL PCR reaction contained 1 µL of DNA template (50 ng), 20 pmol of each primer, 2 to 4 mM MgCl_2_, 2.5 µl of thermophilic 10× reaction buffer without MgCl_2_ (500 mM KCl, 100 mM Tris-HCl [pH 9 at 25°C] and 1% Triton X-100), 2 mM of all four deoxynucleoside triphosphates (dNTPs, Amersham BioSciences), 0.3 µL of *Taq* and 0.2 µL *Pfu* DNA polymerase. The resulting product was analysed by gel electrophoresis and gel purified. In the second round of PCR, each flank was fused with one half of the selectable marker (LF/IL and RF/LV) using nested primers Agl1-nesF/IL split and Agl1-NesR/LV split. The conditions were set as initial denaturation at 94°C for 5 min, followed by 35 cycles of PCR cycling parameters, 94°C for 30 sec, 62°C for 30 sec, 72°C for 3 min, followed by final extension at 72°C for 10 min. The resulting product was analysed by gel electrophoresis and gel purified ready for fungal transformation. Homologous recombination between the overlapping regions of the selectable marker and chromosomal DNA results in targeted deletion of the gene [25]. The transformants were screened by DNA blot analysis. The sequences of the primers used in the study are shown in Table S1.

### Enzyme activity assays

Loss of amyloglucosidase activity in Δ*agl1* strains, and loss of glycogen phosphorylase activity in Δ*gph1* strains, was confirmed enzymatically using proteins extracted from Δ*agl1* and Δ*gph1* strains, compared to Guy11. Strains were grown in GMM for 16 hr, followed by freeze drying and sample preparation, as described previously [12]. Briefly, 10 mg of dried mycelia per strain, in triplicate, was finely ground and re-suspended in 1 ml of sterile distilled H_2_O. Each sample was snap-freezed in liquid nitrogen to break the mycelial cells and liberate total cell protein, and centrifuged for 3 mins. After centrifugation, samples were maintained on ice. 50 µl of this solution, containing total cell protein, was aspirated, in triplicate, from each sample and added to 1 ml of each enzyme assay. All enzymatic assays were performed at 22°C. All assay components were purchased from Sigma. Enzyme activities were determined spectrophotometrically in triplicate and are given as the concentration of product formed in one minute by total cell protein from 1 mg of mycelium. Amyloglucosidase activity [52] was determined by incubating 50 µl of each total cell protein solution with a 1% starch solution, excess purified hexokinase and excess purified glucose 6-phosphate dehydrogenase. Agl1 in the protein sample liberates glucose from starch, which is phosphorylated by hexokinase and the resulting glucose 6-phosphate used to generate NADPH from NADP using glucose 6-phosphate dehydrogenase. The rate of increase in NADPH as the enzyme assay progressed, measured spectrophotometrically at A_340_ and compared to the rate of NADPH production in assays containing no starch or boiled mycelial protein extract, was measured to calculate the activity of Agl1 in each sample. For glycogen phosphorylase activity [52], 50 µl of protein extract from each sample was added to 1 ml of enzyme assay, in triplicate. Each assay contained 4% glycogen, excess purified phosphoglucomutase and excess purified glucose 6-phosphate dehydrogenase. Gph1 liberated glucose-1-phosphate from glycogen, which was converted to glucose-6-phosphate by phosphoglucomutase. Glucose 6-phosphate dehydrogenase converted NADP to NADPH using glucose 6-phosphate, and the rate of increase in NADPH as the enzyme assay progressed, measured at at A_340_ and compared to control reactions lacking glycogen or protein extract, was used to calculate the activity of Gph1in each sample. Protocols were obtained from the Sigma website (www.sigma.com). Trehalose concentrations were measured as described previously [12]. Briefly, samples were treated with trehalase to liberate glucose from trehalose, and glucose was measured using excess purified hexokinase to generate glucose 6-phosphate, which was used by excess glucose 6-phosphate dehydrogenase to generate NADPH from NADP. The amount of NADPH generated in samples treated with trehalase, compared to the same samples not treated with trehalase, was measured at A_340_ and the amount of trehalose in each sample was calculated.

### Generation of Agl1:sGFP fusion plasmid construct

The *AGL1* gene was amplified from genomic DNA of isogenic wild type strain Guy11 with primers Agl1-Pro-U/Agl1-Fus-D, the 2.8 kb acetolactate synthase gene allele (*ILV1*), conferring resistance to sulfonylurea, was amplified with primers Sur-U/Sur-D from pCB1532 [36] and 1.4 kb sGFP:trpC was amplified with primers GFP-U/TrpC-D from pNOX1sGFP. The sequences of the primers are shown in Supplemental Table S1. The PCR fragments were transformed with *Spe*I/*Hin*dIII digested pNEB1284 yeast into *S. cerevisiae*. The gene fusion was constructed by yeast gap repair cloning, based on homologous recombination in yeast [53]. Resulting plasmid was transformed into the isogenic wild type strain Guy11.

### Generation of Gph1:sGFP fusion plasmid construct

A 4.1 kb amplicon consisting of the *M. oryzae GPH1* promoter and open reading frame was PCR amplified from Guy11 genomic DNA with primers Gph1-P-SacII and Gph1-SpeI-D and cloned into pGEM®-T (Promega). The fragment was excised with *Sac*II/*Spe*I restriction sites and cloned into pCB1532, consisting of sulfonylurea resistance gene allele (acetolactate synthase or *ILV1*), to generate pGPH1. A 1.4 kb fragment of the *sGFP:trpC* cassette (Chiu *et al.*, 1996) was PCR amplified using primers GFP-SpeI-U and TrpC-ClaI-D (Supplemental table S1) and subsequently cloned into pGPH1 with *Spe*I restriction sites to create pGPH1sGFP fusion. The fusion plasmid was transformed in isogenic Guy11 of *M. oryzae*.

### Phenotypic analysis of mutants

To determine vegetative growth rates colony diameter was measured after 12 days growth on complete medium [41]. The frequency and nature of conidial germination and appressorium formation was determined microscopically by counting the numbers of germ tubes and/or appressoria formed from conidia after incubation on hydrophobic plastic cover slips for varying lengths of time.

Glycogen mobilisation was visualised microscopically as described above.

The effect of each gene deletion on fungal pathogenicity was determined by spraying 5×10^4^ spores/ml onto susceptible CO-39 rice and susceptible Golden Promise barley lines as described previously [41].

To investigate appressorium turgor generation an incipient cytorrhysis (cell collapse) method [54], [55] was used. Conidia at a concentration of 2×10^4^ ml^−1^ were incubated on hydrophobic plastic coverslips to induce appressorium formation. The surrounding water was then removed and replaced with an equal volume of glycerol solution ranging in concentration from 0.5 to 5.0 M. After 10 minutes incubation the number of appressoria that had collapsed was recorded. Cuticle penetration by appressoria was investigated using onion epidermal peels as described by Chida and Sisler (1987). Conidia at 5×10^4^ spores/ml were allowed to germinate on onion epidermis and the rate of successful appressorium-mediated penetration of the epidermis assayed by light microscopy. All experiments were performed in triplicate.

Appressorium-mediated penetration of rice leaf sheaths was assessed using a procedure based on that of Kankanala *et al*, 2007 [28]. A conidial suspension at a concentration of 1×10^5^ spores mL^−1^ was harvested in 0.25% gelatin and inoculated onto leaf sheaths above the mid vein. The inoculated sheaths were left in the moist petri dishes for 24, 36 and 48 h in a horizontal position to keep the suspension stayed on the mid vein section. When ready for microscopy, the sheaths were hand trimmed to remove the sides and bottom surface to expose a single cell epidermal layer of the mid vein (2–3 cell thick). This epidermal layer was mounted on a glass slide and observed using the Zeiss LSM510 Meta confocal-light scanning microscope (CLSM) system.

### Targeted gene replacement of *GSN1* and *NMR3*

Targeted gene replacement of *GSN1* and *NMR3* was carried out in Guy11 and Δ*agl1*Δ*gph1*, respectively, using split marker strategy as explained earlier, where *GSN1* was replaced with the hygromycin B resistance selectable marker *hph* and *NMR3* was replaced with the bialaphos resistance selectable marker *bar*. The sequences of the primers used in the study are shown in Supplemental table S1. Following transformation, transformants were screened in the presence of hygromycin B (200 mg mL^−1^) for hygromycin resistance and glufosinate ammonium (100 mg mL^−1^) for baialaphos resistance. Gene replacements were confirmed by DNA blot analysis.

### Gene expression analysis

Quantitative RT-PCR was used to characterise the gene expression of *TPS1* and *TPS3* in Guy-11, Δ*agl1*, Δ*gph1* and Δ*agl1*Δ*gph1*. To analyse gene expression of mycelia grown in complete medium, the isogenic wild type Guy-11, Δ*agl1*, Δ*gph1* and Δ*agl1*Δ*gph1*was inoculated into CM 48 hr followed by RNA extraction [41], cDNA synthesis and QRT-PCR analysis. cDNA synthesis was performed using AffinityScript QPCR cDNA synthesis kit (Stratagene), following the manufacturer's protocol. Gene expression analysis was performed using the Brilliant Green QPCR Master Mix kit from Stratagene and the MX3005P instrument (Stratagene) following the manufacturer's protocol. Expression of *TPS1*, *TPS3* and *RSY1* was analysed using the primers Tps1-qRT-5′ (5′-CTTATCGTCAACCCCTGGAAC-3′) and Tps1-3′ (5′-TCCCTCCGTCTTGTTGTCGC-3′), Tps3-qRT-5′ (5′-CACATCAACGACGCCTGCGA-3) and Tps3-3′ (5′-ATGTAGCTCTTGCCTCTGTGTT-3′) and Rsy1-qRT-5′ (5′- CTACCACCAGCTGCGCGTC-3′) and Rsy1-qRT-3′ (5′-TTATTTGTCGCCAAAGGTCTCC-3′). Expression was normalised relative to β-tubulin (MGG_00604.6) gene expression using the primers QRT.PCR.BTubF (CGCGGCCTCAAGATGTCGT) and QRT.PCR.BTubR (GCCTCCTCCTCGTACTCCTCTTCC). PCR conditions were 94°C for 30 seconds, 62°C for 30 seconds and 72°C for 30 seconds.

## Supporting Information

Figure S1 Alignment of the *M. oryzae* Agl1 protein with amyloglucosidase proteins. The predicted *M. oryzae AGL1* gene product was aligned with: Gdb1p from *Saccharomyces cerevisiae* (GenBank accession NP_015510) and hypothetical Agl1 protein from *Neurospora crassa* (EAA35160). Identical residues are indicated on a black background. Conserved residues are indicated on a light grey background and similar residues on a dark grey background. Gaps in the alignments are indicated by dashes. Sequences were aligned using the ClustalW program (Thompson *et al.*, 1994) and shaded using BoxShade v 2.01 (http://www.ch.embnet. org/software/BOX_form.html).
(DOC)

Figure S2 Alignment of the *M. oryzae* Gph1 protein with other glycogen phosphorylase proteins. The predicted *M. oryzae* Gph1 gene product was aligned with: Gph1p from *Saccharomyces cerevisiae* (GenBank accession NP_015486) and a hypothetical Gph1 protein from *Neurospora crassa* (EAA32930). Identical residues are indicated on a black background. Conserved residues are indicated on a light grey background and similar residues on a dark grey background. Sequences were aligned using the ClustalW program (Thompson et al., 1994) and shaded using BoxShade v 2.01 (http://www.ch.embnet.org/software/BOX_form.html).
(DOC)

Figure S3 Targeted gene deletion of *AGL1* and *GPH1* in *M. oryzae* Guy11. **A**. Organization of *AGL1* showing restriction sites and orientation of the coding region. **B**. Targeted gene replacement vectors for *AGL1* gene deletion showing and generation of null mutants. **C**. DNA gel blot analysis of *M. oryzae* Δ*agl1* mutants. DNA was digested with *Nhe*I, fractionated, blotted and probed with a 2.8 kb *Hin*dIII-*Hin*dIII *AGL1* deleted fragment, a 1.7 kb *Hin*dIII-*Hin*dIII *AGL1* promoter fragment or the 1.4 kb *Hph* cassette. Lane 1, Guy11; Lane 2–5, Δ*agl1* mutants; Lane 6 & 7, ectopic *agl1* transformants. **D**. Organisation of the *GPH1* locus **E**. Targeted gene replacement vectors for *GPH1* gene deletion showing and generation of null mutants **F**. DNA gel blot analysis of *M. oryzae* Δ*gph1* mutants. DNA was digested with *Sph* I, fractionated, blotted and probed with a 2.2 kb *GPH1* deleted fragment, a 1.4 kb *GPH1* promoter fragment or the 1.4 kb *Hph* cassette. Lane 1, Guy11; Lane 2, 4 & 5, Δ*gph1* mutants; Lane 3 & 6, ectopic *gph1* transformants.
(PDF)

Figure S4 Targeted gene disruption of the *AGL1* gene in a Δ*gph1* mutant of *M. oryzae*. **A**. Schematic representation of *AGL1* locus showing the restriction sites and orientation of the coding region. DNA was isolated and checked for successful targeted homologous recombination event place by **B**. PCR analysis. Two primers, Agl1-50.1 and Agl1-30.1 were used to detect size difference between ectopic transformants and potential Δ*agl1*Δ*gph1* double mutants. L = 1 kb plus DNA Ladder (Invitrogen), G = genomic DNA from Guy11, lanes 1–10 genomic DNA from putative Δ*agl1*Δ*gph1* transformants. Lanes 6, 7, 10 = putative Δ*agl1*Δ*gph1* double mutants. **C**. Putative Δ*agl1*Δ*gph1* mutants were verified with Southern blot hybridization analysis. Lane G = Guy11; Lane g = Δ*gph1*; lane 2 = ectopic transformant 2 ; Lanes 6, 7, 10 = putative Δ*agl1*Δ*gph1* double mutants. The blot was probed with the deleted region of *AGL1*. **D**. Total RNA was isolated and cDNA synthesized. The *AGL1* 5′ coding region (789 bp) was amplified in a non-quantitative RT-PCR reaction. All three analyses confirmed successful gene disruption of the *AGL1* gene in a Δ*gph1* background mutant. Transformant 6 (AG-6) was selected for further phenotypic analysis and hereafter is referred to as Δ*agl1*Δ*gph1*. L = 1 kb plus DNA Ladder, G = Guy11 (Wild Type), g = Δ*gph1*, a = Δ*agl1*, gDNA = genomic Guy11 DNA.
(PDF)

Figure S5 Complementation of Δ*agl1* and Δ*gph1* mutants of *M. oryzae* restores growth on starch. Full length *AGL1* and *GPH1* genes, under control of their native promoters were transformed into Δ*gph1* and Δ*gph1* mutants respectively and transformants selected. Plate assays were carried out on minimal medium with starch as sole carbon source. Reintroduction of each gene restored their ability to grow on starch.
(PDF)

Figure S6 Amino acid alignment of the predicted *M. oryzae* Gsn1 protein with reported glycogen synthases. The predicted *M. oryzae GSN1* (MGG_07829.6) amino acid sequence was aligned with translated products of *Homo sapiensf*, Gys1 & Gys2, and *Saccharomyces cerevisiae*, Gsy1 (YFR015C) & Gsy2 (YLR258W). Sequences were identified from NCBI and SGD database (http://www.yeastgenome.org/), aligned using ClustalW (Thompson *et al.*, 1994) and shaded using GeneDoc Version 2.6.002. Residues within black are identical among all listed proteins, conserved changes are shown in grey and those in white background do not show any similarity. Conserved putative serine or threonine residues that are candidates for post-translational phosphorylation are shown in blue.
(DOC)

Figure S7 Targeted gene deletion of *GSN1* in *M. oryzae* Guy11. **A**. Diagrammatic representation of *GSN1* locus. **B**. DNA was isolated, digested by *Stu*I and fractionated in a 0.8% agarose gel. The gel was processed by Southern blot analysis and probed with a 2.35 kb fragment of the *GSN1* coding region to show presence or absence of the coding region A 0.96 kb fragment of the upstream flanking region was also used as a probe to show a restriction fragment length polymorphism shown in the bottom panel. The transformants G3, G10, G12 and G13 are putative Δ*gsn1* mutants. L = 1 kb plus DNA Ladder (Invitrogen). G2 & G4 = ectopic transformants.
(PDF)

Figure S8 Targeted gene deletion of *NMR3* in a Δ*agl1*Δ*gph1* mutant of *M. oryzae*. **A**. Organization of the *NMR3* locus to show *Pst* I restriction sites and schematic representation showing mechanism of *NMR3* gene deletion using split marker strategy. DNA was isolated from Δ*agl1*Δ*gph1* and putative Δ*agl1*Δ*gph1*Δ*nmr3* transformants and digested with *Pst* I. The digested DNA was fractionated in a 0.8% agarose gel and transferred to Hybond-N. **B**. The membrane was probed with a 1 kb 5′ flanking DNA fragment to confirm Δ*agl1*Δ*gph1*Δ*nmr3* mutants on the basis of a restriction fragment length polymorphism. Transformant 10 was selected for further phenotypic analysis and hereafter named Δ*agl1*Δ*gph1*Δ*nmr3*. WT = Guy11, 1–11 = putative Δ*agl1*Δ*gph1*Δ*nmr3* transformants and AG6 = Δ*agl1*Δ*gph1*.
(PDF)

Table S1 Oligonucleotide primers used in this study.
(DOCX)
