# Supplementary material for: Glycogen Metabolic Genes Are Involved in Trehalose-6-Phosphate Synthase-Mediated Regulation of Pathogenicity by the Rice Blast Fungus Magnaporthe oryzae
Source: PLoS Pathog. 2013 Oct 3;9(10):e1003604. doi: 10.1371/journal.ppat.1003604 (PMC3789717; doi:10.1371/journal.ppat.1003604)
Supplement: Figure S1 — Alignment of the M. oryzae Agl1 protein with amyloglucosidase proteins. The predicted M. oryzae AGL1 gene product was aligned with: Gdb1p from Saccharomyces cerevisiae (GenBank accession NP_015510) and hypothetical Agl1 protein from Neurospora crassa (EAA35160). Identical residues are indicated on a black background. Conserved residues are indicated on a light grey background and similar residues on a dark grey background. Gaps in the alignments are indicated by dashes. Sequences were aligned using the ClustalW program (Thompson et al., 1994) and shaded using BoxShade v 2.01 (http://www.ch.embnet. org/software/BOX_form.html). (DOC) [file ppat.1003604.s001.doc]

AGL1 *M.grisea* 1 .....MISNEVYYLPLNDDGSPQVQGEY............IYLAPKSNEPFTIRFAIEGT
AGL1 *N.crassa* 1 MIPRTMVSNEVYLLPLKDDGSPDVPGEY............IYIAPKSKDPVTIRFAIEGT
GDB1 *S.cerevisiae* 1 .......MNRSLLLRLSDTGEPITSCSYGKGVLTLPPIPLPKDAPKDQPLYTVKLLVSAG

AGL1 *M.grisea* 44 SSICRQGSLWVNIP.EPGVEFRRDQFREFKLVP...............DFNRTIEISIPV
AGL1 *N.crassa* 49 SSICSHGSLWVNIP.EHGEQFQRNKFREFKLVP...............DFNRTLEISIPI
GDB1 *S.cerevisiae* 54 SPVARDGLVWTNCPPDHNTPFKRDKFYKKIIHS...............SFHEDDCIDLNV

AGL1 *M.grisea* 88 HLPGAYAFYTTYAKLPELEQTAG.ADSSPSSTDKNKTQLYYIDVAPRLQLD....GRPLP
AGL1 *N.crassa* 93 YEAGAYAFYTTYAELPDLASNLVNADGTVTKATQKKTPLYYIDVAPRLSLD....GQPLP
GDB1 *S.cerevisiae* 99 YAPGSYCFYLSFRND...............NEKLETTRKYYFVALPMLYIN....DQFLP

AGL1 *M.grisea* 143 LPALSIFSVISKFMGKYPNDWERHLRGISDRGYNMIHFTPLQTRGTSNSPYSLYDQLSWD
AGL1 *N.crassa* 149 LPALSIFSIISKFMGKYPTDWERHLRGISDRGYNMIHFTPLQVRGASNSPYSLYDQLGWD
GDB1 *S.cerevisiae* 140 LNSIALQSVVSKWLG...SDWEPILSKIAAKNYNMVHFTPLQERGESNSPYSIYDQLQFD

AGL1 *M.grisea* 203 PECFPEGEK.....DIKKLVDSLEKNHSLLSLTDIVLNHTADNSEWLLEHPEAGYNLTTA
AGL1 *N.crassa* 209 PACFPAGEP.....DVQKMVESLEKNHSLLSLTDIVLNHTAHNSEWLLEHPEAGYNLTTA
GDB1 *S.cerevisiae* 197 QEHFKSPE......DVKNLVEHIHRDLNMLSLTDIVFNHTANNSPWLVEHPEAGYNHITA

AGL1 *M.grisea* 258 PWLELPFLIDTKLLELGFNLAK.....LGLPTEVKSEADVLAIMDAVKKEVISAIRMWEY
AGL1 *N.crassa* 264 PWLESAYLLDTKLLELGTRLEE.....LGLPTELKDVDDLVKIMDAIKKEVIAEIRLWEY
GDB1 *S.cerevisiae* 251 PHLISAIELDQELLNFSRNLKS.....WGYPTELKNIEDLFKIMDGIKVHVLGSLKLWEY

AGL1 *M.grisea* 313 YVVDVESNAEVAAEAWAAGKG...SFPDGSLGTEG.PAGLKSASLSERAQFVIKYGLSGT
AGl1 *N.crassa* 319 YTLDVERDADAAVKSWAANDI...DFPQASVGAGG.IDSLHSATPKEQADFLIQHGLQNM
GDB1 *S.cerevisiae* 306 YAVNVQTALRDIKAHWNDESNESYSFPENIKDISSDFVKLASFVKDNVTEPNFGTGERNS

AGL1 *M.grisea* 369 DRMGERFRRKVVPEVAAGVLDALLGRPEGHQGPDSGAARQKMIEILQAVNVPFYEEYDKD
AGL1 *N.crassa* 375 DYL.................................A.RTKIVEILEIVNVPFYKEYDDE
GDB1 *S.cerevisiae* 366 NRIN............VPKFIQLLKLINDGGSDDSESSLATAQNILNEVNLPLYREYDDD

AGL1 *M.grisea* 429 SAEILEQTFNRIKYVRLDEHGPKLGPINEANPLVETYFTRLPRNEK.......TAKHKKE
AGL1 *N.crassa* 401 VAEILQQLFNRIKYVRLDDHGPKLGPINAENPLIETYFTRLPVNEK.......TKKHKKE
GDB1 *S.cerevisiae* 414 VSEILEQLFNRIKYLRLDDGGPKQGPVTVDVPLTEPYFTRFKGKD........GT.....

AGL1 *M.grisea* 482 DLVLVNNGWVWG.GNALVDNAG...PDSRVYLRREVIVWGDCVKLRYGAGPEDSPYLWDR
AGL1 *N.crassa* 454 DLVLANNGWVWG.GNALVDNAG...PDSRVYLRREVIVWGDCTKLRYGSGPEDSPWLWEH
GDB1 *S.cerevisiae* 461 DYALANNGWIWN.GNPLVDFAS...QNSRAYLRREVIVWGDCVKLRYGKSPEDSPYLWER

AGL1 *M.grisea* 538 MTKYSRMLAKYFAGFRIDNAHSTPIHVAEHILDEARRVRPDLYVCGELFTGSEEMDYVFV
AGL1 *N.crassa* 510 MTKYARMLAKYFAGFRIDNCHSTPLHVAEHILDEARRVRPDLYVVAELFTGSEEMDYVFV
GDB1 *S.cerevisiae* 517 MSKYIEMNAKIFDGFRIDNCHSTPIHVGEYFLDLARKYNPNLYVVAELFSGSETLDCLFV

AGL1 *M.grisea* 598 KRLGLSSLIREAMQAWSTGELSRLVHRHGGRPIGSFEVDEVSRGDASPTSPHPQSPQVNG
AGL1 *N.crassa* 570 KRLGISALIREAMQAWSTGELSRLVHRHGGRPIGSFEVDEVSSNEGRSSS........IS
GDB1 *S.cerevisiae* 577 ERLGISSLIREAMQAWSEEELSRLVHKHGGRPIGSYKFVPMDDFSYPADINLNEEHCFND

AGL1 *M.grisea* 658 GTTDEMWSSREIIR..RIKPSPVQALFMDCTHDNEVPAQKRDARDTLPNAALVSMCASAT
AGL1 *N.crassa* 622 GTNGDGVYTREVIR..RIRPVPVQALFMDCTHDNEVPAQKRDARDTLPNAALVAMCASAT
GDB1 *S.cerevisiae* 637 SNDNSIRCVSEIMIPKILTATPPHALFMDCTHDNETPFEKRTVEDTLPNAALVALCSSAI

AGL1 *M.grisea* 716 GSVMGYDEIYPKLVDLVNETRLYTSESSAR...EVKVGSGKGGIGGLKKLMNQIHTLMGK
AGL1 *N.crassa* 680 GSVMGYDEIYPKLVDLVGETRLYTSEASKA...PVKTGEGKDGIAGVKKLLNQIHTLMGM
GDB1 *S.cerevisiae* 697 GSVYGYDEIFPHLLNLVTEKRHYDIS........TPTGSPSIGITKVKATLNSIRTSIGE

AGL1 *M.grisea* 773 DGYD....ETHIHHEDQYITVHRVHPQSRKGYFLIAHTAYP..GYGNGNGAFNPVHLTGT
AGL1 *N.crassa* 737 DGYD....ETHIHHEDEYVTVHRVHQESRKGYFLIAHTAFP..GYGNGNGAFNPVHLTGT
GDB1 *S.cerevisiae* 749 KAYDIEDSEMHVHHQGQYITFHRMDVKSGKGWYLIARMKFS..DNDDPNETLPPVVLNQS

AGL1 *M.grisea* 827 KVRHLGSWTLEVDDSE.........EARKNVLEDKKHLRGLPSRVIDLPG.....IRMEV
AGL1 *N.crassa* 791 KARHLGSWMLEVDASK.........EAVEEVLSDKKHLRGLPSRLVGLPG.....VRMEV
GDB1 *S.cerevisiae* 807 TCSLRFSYALER..............VGDEIPNDDKFIKGIPTKLKELEG.....FDISY

AGL1 *M.grisea* 873 KGSD..TVITVRDRFPPGSIALFETYIPAAEHSAG.........LDTYVTSQAQDAFAG.
AGL1 *N.crassa* 837 KGQD..TIITVREKFPPGSIALFETWIPAAEHSSG.........LDNFVTSGAKAAMDE.
GDB1 *S.cerevisiae* 848 DDSKKISTIKLPNEFPQGSIAIFETQQNGVDES...........LDHFIRSGALKATSS.

AGL1 *M.grisea* 920 .............LNLIDMNFMMYRCEAEERDWSGGSDGVYGIPGHGNLVYAGLQGWWSL
AGL1 *N.crassa* 884 .............LDLVDLNFLLYKCEPEERDASEGQDGTYDIPGHGKIVYAGLQGWWSI
GDB1 *S.cerevisiae* 895 .............LTLESINSVLYRSEPEEYDVSAGEGGAYIIPNFGKPVYCGLQGWVSV

AGL1 *M.grisea* 968 LEGIIRDNNLAHPLCQNLREGQWALDFIIGRLERASKTPEFSRLEKPTRWLKDRFDAIRG
AGL1 *N.crassa* 932 LKDVIKDNNLAHPLCQHLRDGQWALDYIVGRLERASKKDDFRRLAKPAQWLKERFDAIRP
GDB1 *S.cerevisiae* 943 LRKIVFYNDLAHPLSANLRNGHWALDYTISRLNYYSDEAGINEVQN...WLRSRFDRVKK

AGL1 *M.grisea* 1028 IPSFLLPRYFALVMRTAYMACFERSVSLMNENVAKGQWFLKSLAMVSTQETGIVKSASL.
AGL1 *N.crassa* 992 IPSFLLPRYFGLVLRTAYNAAFERGISLMNNNVIKGQWFLQSLAMVSVQMTGLVKSASL.
GDB1 *S.cerevisiae*  1000 LPSYLVPSYFALIIGILYGCCRLKAIQLMSRNIGKSTLFVQSLSMTSIQMVSRMKSTSI.

AGL1 *M.grisea* 1086 ..................YPDHLVPSLAAGLPHFAVDWARCWGRDIFISLRGLFLGTGRF
AGL1 *N.crassa* 1050 ..................YPNRLVPSLAAGLPHFAVEWARCWGRDVFISLRGLYLGTGRY
GDB1 *S.cerevisiae* 1058 ..................LPGENVPSMAAGLPHFSVNYMRCWGRDVFISLRGMLLTTGRF

AGL1 *M.grisea* 1129 DEAREHILAFASVLKHGMIPNLLGAGRTPRYNARDSIWFFLQCIQDYTRLVPNGLSLLDE
AGL1 *N.crassa* 1093 AEAREHIHAFASVLKHGMIPNLLGSGNNPRYNSRDSVWFFLQCIQDYTRLVPDGLSILDD
GDB1 *S.cerevisiae* 1101 DEAKAHILAFAKTLKHGLIPNLLDAGRNPRYNARDAAWFFLQAVQDYVYIVPDGEKILQE

AGL1 *M.grisea* 1189 KVKRRFLPYDDTYFDAEDSRAYSTQSTIRDIIQEALQRHASGMSFREANAGPSLDMQMSD
AGL1 *N.crassa* 1153 KVKRRFLPYDDTYFDVDDERAYSKESTIAEIIQEVFQRHAEGMKYREANAGPNLDMQMSD
GDB1 *S.cerevisiae* 1161 QVTRRFP.LDDTYIPVDDPRAFSYSSTLEEIIYEILSRHAKGIKFREANAGPNLDRVMTD

AGL1 *M.grisea* 1249 KGFDLEVKVDWDTGFVLGGNQFNCGTWMDKMGESEKAGSKGVPGTPRDGAAVEITGLMYS
AGL1 *N.crassa* 1213 AGFNQEIKVDWSNGFIFGGNQANCGTWMDKMGESERAGSKGVPGTPRDGAAVEITGMLYS
GDB1 *S.cerevisiae* 1220 KGFNVEIHVDWSTGLIHGGSQYNCGTWMDKMGESEKAGSVGIPGTPRDGAAIEINGLLKS

AGL1 *M.grisea* 1309 TVKWVAKLHSSGEFPYSGVSKAAVGPDQDPKSVNVDVSYADWAELVKSNFERCYFVPLDP
AGL1 *N*.*crassa* 1273 NLKWAASLNEAGKFKHSSVRKADG..........TEITLKDWAALIKDNFERCYFVPISP
GDB1 *S.cerevisiae* 1280 ALRFVIELKNKGLFKFSDVETQDGG..........RIDFTEWNQLLQDNFEKRYYVPEDP

AGL1 *M.grisea* 1369 SEDSKYDVNPSV.INRRGIYKDLYRSGKEYEDYQLRPNFPIAMTVAPDLFDEAHAMHALC
AGL1 *N.crassa* 1323 DEDSKYDVNPAI.INRRGIYKDLYRSGKEYEDYQLRCNFPIAMTCAADLFVPEHAMHALW
GDB1 *S.cerevisiae* 1330 SQDADYDVSAKLGVNRRGIYRDLYKSGKPYEDYQLRPNFAIAMTVAPELFVPEHAIKAIT

AGL1 *M.grisea* 1428 IADQVLRGPQGMATLDPADLNYRPYYVNSEDSTDFATSKGRNYHQGPEWLWPTGFFLRAL
AGL1 *N.crassa* 1382 VADAALRGPTGMATLDPSDMNYRPYYNNSEDSDDFATSKGRNYHQGPEWLWPTGFFLRAL
GDB1 *S.cerevisiae* 1390 IADEVLRGPVGMRTLDPSDYNYRPYYNNGEDSDDFATSKGRNYHQGPEWVWLYGYFLRAF

AGL1 *M.grisea* 1488 LKFLLKRT.HSAEDGESAA..TEAFQQVTRRLMGCKAMIKESPWAGLQELTQKDGGFCGD
AGL1 *N.crassa* 1442 LKFDLMR...RGRDDAEGR..TEAFQQVTRRLIGCKEMIQRSPWAGLTELTNKNGEECHD
GDB1 *S.cerevisiae* 1450 HHFHFKTS.PRCQNAAKEKPSSYLYQQLYYRLKGHRKWIFESVWAGLTELTNKDGEVCND

AGL1 *M.grisea* 1545 SCPTQAWSASCLIDLYMDADEEQARMQKGVGVNGSSQKN 1583
AGL1 *N.crassa* 1497 SSPTQAWSAGCLIDLYMDAAEEQAKLEK....HSLPLR. 1530
GDB1 *S.cerevisiae* 1509 SSPTQAWSSACLLDLFYDLWDAYEDDS............ 1535

**Figure S1**
