# Supplementary material for: Glycogen Metabolic Genes Are Involved in Trehalose-6-Phosphate Synthase-Mediated Regulation of Pathogenicity by the Rice Blast Fungus Magnaporthe oryzae
Source: PLoS Pathog. 2013 Oct 3;9(10):e1003604. doi: 10.1371/journal.ppat.1003604 (PMC3789717; doi:10.1371/journal.ppat.1003604)
Supplement: Figure S2 — Alignment of the M. oryzae Gph1 protein with other glycogen phosphorylase proteins. The predicted M. oryzae Gph1 gene product was aligned with: Gph1p from Saccharomyces cerevisiae (GenBank accession NP_015486) and a hypothetical Gph1 protein from Neurospora crassa (EAA32930). Identical residues are indicated on a black background. Conserved residues are indicated on a light grey background and similar residues on a dark grey background. Sequences were aligned using the ClustalW program (Thompson et al., 1994) and shaded using BoxShade v 2.01 (http://www.ch.embnet.org/software/BOX_form.html). (DOC) [file ppat.1003604.s002.doc]

GPH1 *M.grisea* 1 MATDKPQRIPMRERRPSSGAPLVDITGSVSPAGVSRPKHKRTLTGFGAQEIKSVEASIPE
GPH1 *N.crassa* 1 MASNTTQRVPLRERRPSVGAPLVDIQGGVAPAGVSRPKHKRTLTGFGPGEIKNVEASIPE
GPH1 *S.cerevisiae* 1 .............MPPASTSTTNDMITEEPTSPHQIPRLTRRLTGFLPQEIKSIDTMIPL

GPH1 *M.grisea* 61 GQRAAWSKHQAKPFQDKEEFENEVVRHIETTLARSMFNCNESAAYSAAGLAFRDRLIMEW

GPH1 *N.crassa* 61 PQRKAWLAHQTSGFKDKDGFETEVVRHVETTLARSMYNCDEQAAYSACSLAFRDRLILEW
GPH1 *S.cerevisiae* 48 KSRALWNKHQVKKFNKAEDFQDRFIDHVETTLARSLYNCDDMAAYEAASMSIRDNLVIDW

GPH1 *M.grisea* 121 NKTQQRQTFEDKKRVYYLSLEFLMGRTLDNAMLNLNLKD....VAKQG.........LSE

GPH1 *N.crassa* 121 NRTQQRQTFADSKRVYYLSLEFLMGRALDNAMLNIGQKD....VAKAG.........LAE
GPH1 *S.cerevisiae* 108 NKTQQKFTTRDPKRVYYLSLEFLMGRALDNALINMKIEDPEDPAASKGKPREMIKGALDE

GPH1 *M.grisea* 168 LGFNVEDIIGEERDAALGNGGLGRLAACFLDSLASLNYPAWGYGLRYRYGIFKQEIVDGY
GPH1 *N.crassa* 168 LGFRIEDVIEQEHDAALGNGGLGRLAACFLDSLASLNYSAWGYGLRYRYGIFKQEIIDGY
GPH1 *S.cerevisiae* 168 GGFKLEDVLDQEPDAGLGNGGLGRLAACFVDSMATEGIPAWGYGLRYEYGIFAQKIIDGY

GPH1 *M.grisea* 228 QVEVPDYWLD.QNLWEFPRHDVTVDIQFYGHVEKSQESSGSKTSANWVGGETVTAIAYDM
GPH1 *N.crassa* 228 QVEVPDYWLD.FNPWEFPRHDVTVDIQFYGHVTKRTDDNG.KTIATWEGGEIVKAVAYDV
GPH1 *S.cerevisiae* 228 QVETPDYWLNSGNPWEIERNEVQIPVTFYGYVDRPEGGKTTLSASQWIGGERVLAVAYDF

GPH1 *M.grisea* 287 PIPGYATPTTNNLRLWSSKAASGEFDFQKFNSGEYESSVADQQRAETISAVLYPNDNLER
GPH1 *N.crassa* 286 PIPGYATPSTNNLRLWSSKAASGEFDFQKFNSGDYENSVADQQRAETISAVLYPNDNLDR
GPH1 *S.cerevisiae* 288 PVPGFKTSNVNNLRLWQARPTT.EFDFAKFNNGDYKNSVPQQQRAESITAVLYPNDNFAQ

GPH1 *M.grisea* 347 GKELRLKQQYFWVAASLYDIVRRFKKSKRAWREFPEQVAIQLNDTHPTLAVVELQRILID
GPH1 *N.crassa* 346 GKELRLKQQYFWVAASLYDIVRRFKKSRRPWKEFPDQVAIQLNDTHPTLAVVELQRILVD
GPH1 *S.cerevisiae* 347 GKELRLKQQYFWCAASLHDILRRFKKSKRPWTEFPDQVAIQLNDTHPTLAIVELQRVLVD

GPH1 *M.grisea* 407 LEGLDWDDAWNIVQSTFGYTNHTVLPEALEKWPVGLIQHLLPRHLQIIYDINLFFLQSVE
GPH1 *N.crassa* 406 LEGLDWEEAWNIVTNTFGYTNHTVLPEALEKWSVPLFQHLLPRHLQLIYDINLFFLQSVE
GPH1 *S.cerevisiae* 407 LEKLDWHEAWDIVTKTFAYTNHTVMQEALEKWPRRLFGHLLPRHLEIIYDINWFFLQDVA

GPH1 *M.grisea* 467 RQFPGDRDLLSRVSIIEEGQT.KMVRMAHLAIIGSHKVNGVAELHSDLIKTTIFRDFVEI
GPH1 *N.crassa* 466 RKFPKDREMLARVSIIEESQP.KMVRMAHLAIVGSHKVNGVAELHSDLIKTTIFKDFVEV
GPH1 *S.cerevisiae* 467 KKFPKDVDLLSRISIIEENSPERQIRMAFLAIVGSHKVNGVVELHSELIKTTIFKDFIKF

GPH1 *M.grisea* 526 FGPDKFTNVTNGITPRRWLHQANPKLSELISTKCGS..YDFLKDLTGLNELEKWVKDEEF
GPH1 *N.crassa* 525 FGPDKFTNVTNGITPRRWLHQANPRLSELISSKTGS..QNFLKDLTELAKIEHYKDDKAF
GPH1 *S.cerevisiae* 527 YGPSKFVNVTNGITPRRWLKQANPSLAKLISETLNDPTEEYLLDMAKLTQLEKYVEDKEF

GPH1 *M.grisea* 584 RKEWAAIKRSNKARLADYIKR.TTGVTVS.....PDALFDVQVKRIHEYKRQQMNIFGVI
GPH1 *N.crassa* 583 RKEWAEIKYANKVRLAKHIKK.TTGVDVN.....PSALFDVQVKRIHEYKRQQMNIFGVI
GPH1 *S.cerevisiae* 587 LKKWNQVKLNNKIRLVDLIKKENDGVDIINREYLDDTLFDMQVKRIHEYKRQQLNVFGII

GPH1 *M.grisea* 638 HRYLALKAMTP.....EEREKQLPR.VSIFGGKAAPGYWMAKQIIHLINSVGAVVNKDED
GPH1 *N.crassa* 637 HRYLTLKSLSP.....EERKKFQPR.VSIFGGKAAPGYWMAKQIIHLINAVGAVVNNDKD
GPH1 *S.cerevisiae* 647 YRYLAMKNMLKNGASIEEVAKKYPRKVSIFGGKSAPGYYMAKLIIKLINCVADIVNNDES

GPH1 M.grisea 692 IGDKLKVVFLEDYNVSKAEMIIPANDLSEHISTAGTEASGTSNMKFVLNGGLIIGTCDGA
GPH1 N.crassa 691 IGDLLKVIFLEDYNVSKAEMIIPASDLSEHISTAGTEASGTSNMKFVLNGGLIIGTCDGA
GPH1 S.cerevisiae 707 IEHLLKVVFVADYNVSKAEIIIPASDLSEHISTAGTEASGTSNMKFVMNGGLIIGTVDGA

GPH1 *M.grisea* 752 N..................IEITREIGENNIFLFGNLAEDVEDLRHAHTYGKTHAIDPEL
GPH1 *N.crassa* 751 NVSNNPLFYAMSSLANPLQIEITREIGEQNIFLFGNLAEDVEDIRHNHTYG.SYTVDPDL
GPH1 *S.cerevisiae* 767 N..................VEITREIGEDNVFLFGNLSENVEELRYNHQYH.PQDLPSSL

GPH1 *M.grisea* 794 LKVFDAIQAGKFG..EPQNFGSLIAAIKDHGDYYLVSDDFSSYLDTHKLVDESYRDQ.EG
GPH1 *N.crassa* 810 VKVFEAIEKGTFG..EPNDFMGMISAVRDHGDFYLVSDDFHSYIETQELVDKAYRDQ.EG
GPH1 *S.cerevisiae* 808 DSVLSYIESGQFSPENPNEFKPLVDSIKYHGDYYLVSDDFESYLATHELVDQEFHNQRSE

GPH1 M.grisea 851 WITKCITSVARMGFFTSDRCINEYAEEIWNIEPLKVGA...... 888
GPH1 N.crassa 867 WITKSIESVARMGFFSSDRCINEYAEGIWNIEPLAVKDQ..... 905
GPH1 S.cerevisiae 868 WLKKSVLSLANVGFFSSDRCIEEYSDTIWNVEPVT......... 902

**Figure S2**
