# Supplementary figures and images for: Glycogen Metabolic Genes Are Involved in Trehalose-6-Phosphate Synthase-Mediated Regulation of Pathogenicity by the Rice Blast Fungus Magnaporthe oryzae
Source: PLoS Pathog. 2013 Oct 3;9(10):e1003604. doi: 10.1371/journal.ppat.1003604 (PMC3789717; doi:10.1371/journal.ppat.1003604)

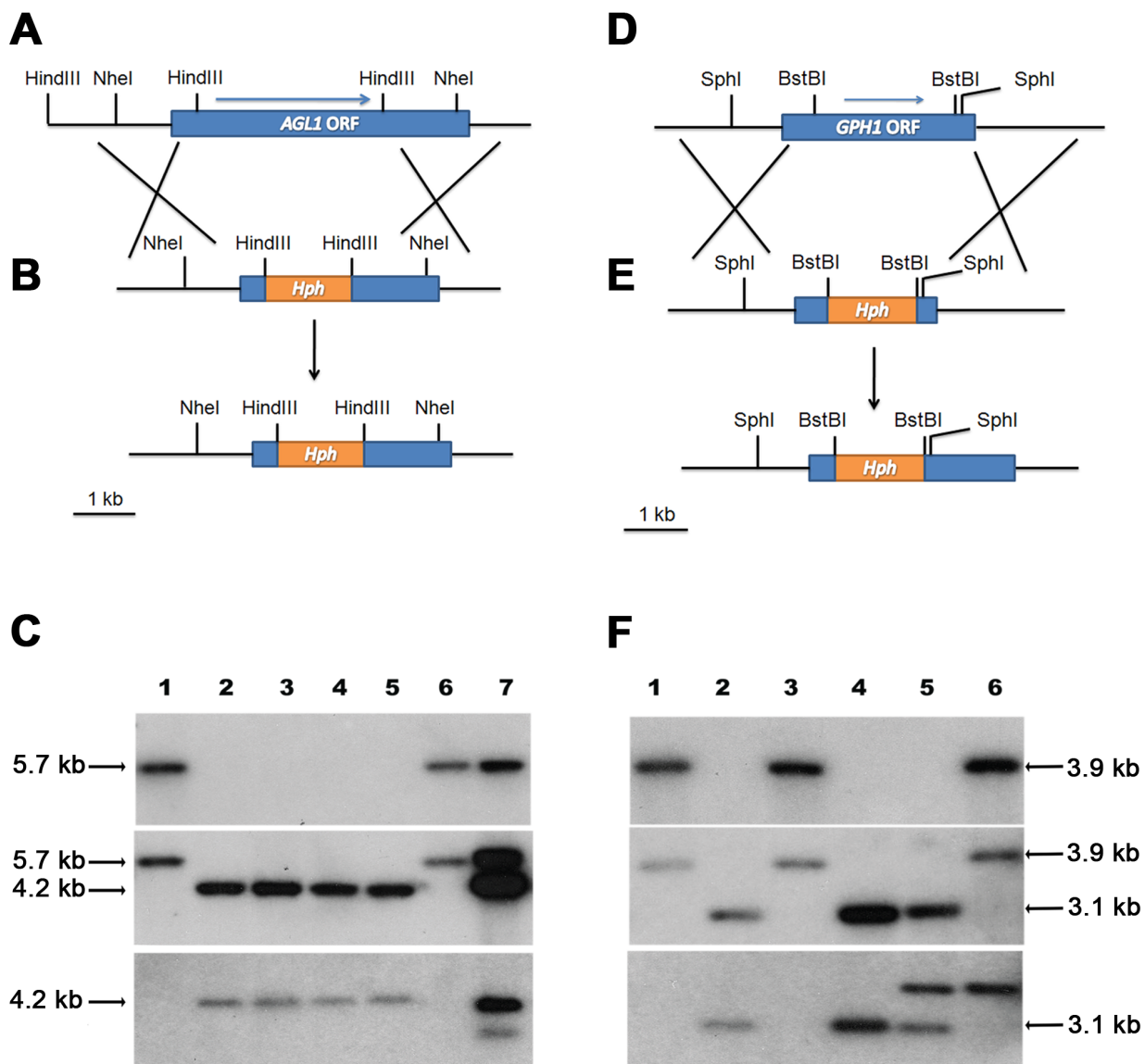

Figure S3

Supplement: Figure S3 — Targeted gene deletion of AGL1 and GPH1 in M. oryzae Guy11. A. Organization of AGL1 showing restriction sites and orientation of the coding region. B. Targeted gene replacement vectors for AGL1 gene deletion showing and generation of null mutants. C. DNA gel blot analysis of M. oryzae Δagl1 mutants. DNA was digested with NheI, fractionated, blotted and probed with a 2.8 kb HindIII-HindIII AGL1 deleted fragment, a 1.7 kb HindIII-HindIII AGL1 promoter fragment or the 1.4 kb Hph cassette. Lane 1, Guy11; Lane 2–5, Δagl1 mutants; Lane 6 & 7, ectopic agl1 transformants. D. Organisation of the GPH1 locus E. Targeted gene replacement vectors for GPH1 gene deletion showing and generation of null mutants F. DNA gel blot analysis of M. oryzae Δgph1 mutants. DNA was digested with Sph I, fractionated, blotted and probed with a 2.2 kb GPH1 deleted fragment, a 1.4 kb GPH1 promoter fragment or the 1.4 kb Hph cassette. Lane 1, Guy11; Lane 2, 4 & 5, Δgph1 mutants; Lane 3 & 6, ectopic gph1 transformants. (PDF) [file ppat.1003604.s003.pdf]

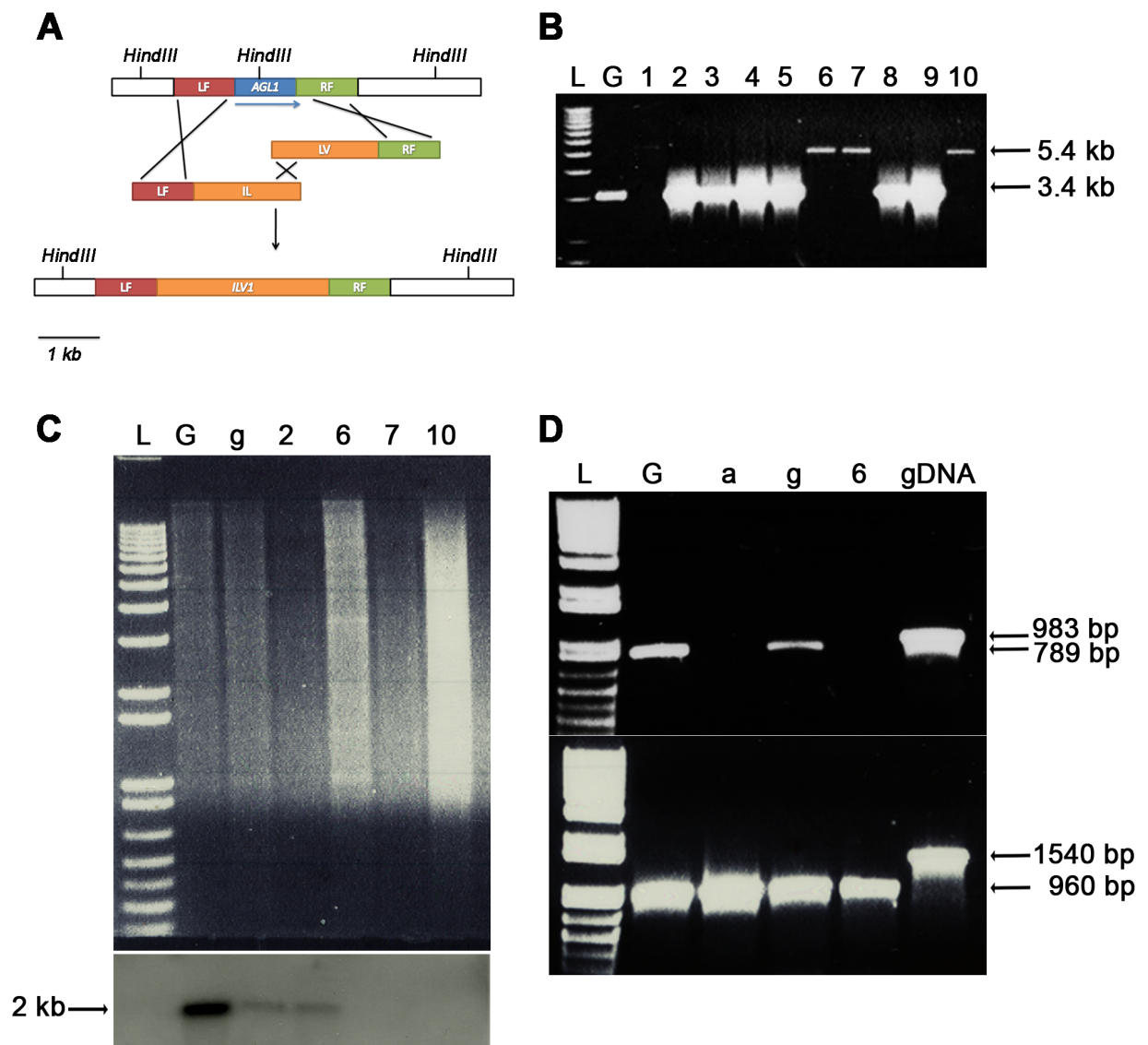

Figure S4

Supplement: Figure S4 — Targeted gene disruption of the AGL1 gene in a Δgph1 mutant of M. oryzae. A. Schematic representation of AGL1 locus showing the restriction sites and orientation of the coding region. DNA was isolated and checked for successful targeted homologous recombination event place by B. PCR analysis. Two primers, Agl1-50.1 and Agl1-30.1 were used to detect size difference between ectopic transformants and potential Δagl1Δgph1 double mutants. L = 1 kb plus DNA Ladder (Invitrogen), G = genomic DNA from Guy11, lanes 1–10 genomic DNA from putative Δagl1Δgph1 transformants. Lanes 6, 7, 10 = putative Δagl1Δgph1 double mutants. C. Putative Δagl1Δgph1 mutants were verified with Southern blot hybridization analysis. Lane G = Guy11; Lane g = Δgph1; lane 2 = ectopic transformant 2 ; Lanes 6, 7, 10 = putative Δagl1Δgph1 double mutants. The blot was probed with the deleted region of AGL1. D. Total RNA was isolated and cDNA synthesized. The AGL1 5′ coding region (789 bp) was amplified in a non-quantitative RT-PCR reaction. All three analyses confirmed successful gene disruption of the AGL1 gene in a Δgph1 background mutant. Transformant 6 (AG-6) was selected for further phenotypic analysis and hereafter is referred to as Δagl1Δgph1. L = 1 kb plus DNA Ladder, G = Guy11 (Wild Type), g = Δgph1, a = Δagl1, gDNA = genomic Guy11 DNA. (PDF) [file ppat.1003604.s004.pdf]

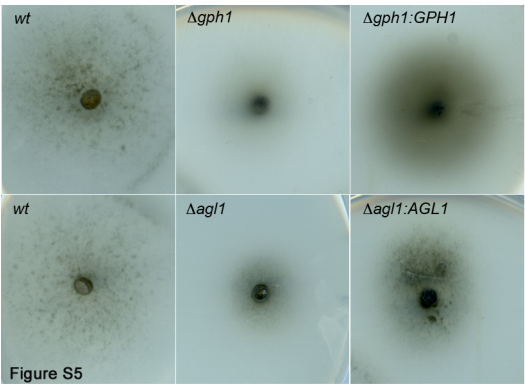

Supplement: Figure S5 — Complementation of Δagl1 and Δgph1 mutants of M. oryzae restores growth on starch. Full length AGL1 and GPH1 genes, under control of their native promoters were transformed into Δgph1 and Δgph1 mutants respectively and transformants selected. Plate assays were carried out on minimal medium with starch as sole carbon source. Reintroduction of each gene restored their ability to grow on starch. (PDF) [file ppat.1003604.s005.pdf]

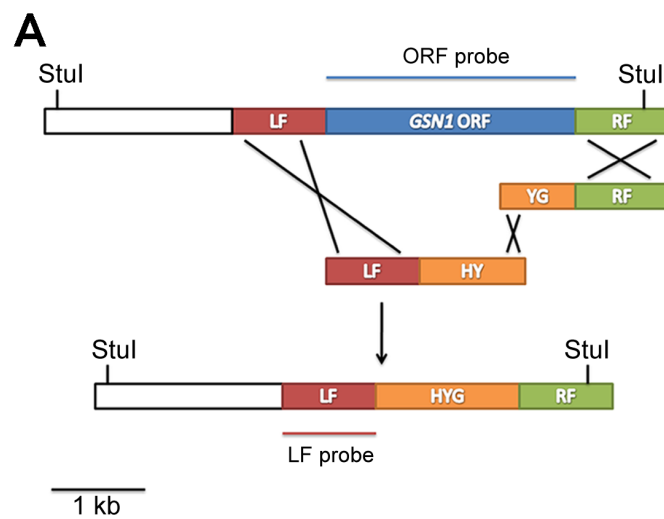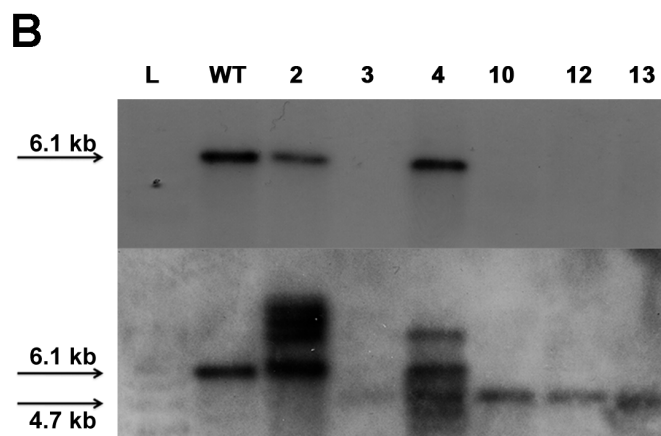

Figure S7

Supplement: Figure S7 — Targeted gene deletion of GSN1 in M. oryzae Guy11. A. Diagrammatic representation of GSN1 locus. B. DNA was isolated, digested by StuI and fractionated in a 0.8% agarose gel. The gel was processed by Southern blot analysis and probed with a 2.35 kb fragment of the GSN1 coding region to show presence or absence of the coding region A 0.96 kb fragment of the upstream flanking region was also used as a probe to show a restriction fragment length polymorphism shown in the bottom panel. The transformants G3, G10, G12 and G13 are putative Δgsn1 mutants. L = 1 kb plus DNA Ladder (Invitrogen). G2 & G4 = ectopic transformants. (PDF) [file ppat.1003604.s007.pdf]

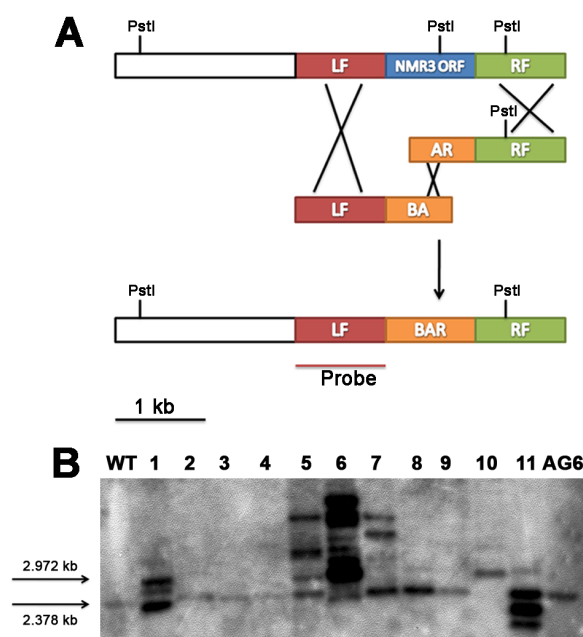

Figure S8

Supplement: Figure S8 — Targeted gene deletion of NMR3 in a Δagl1Δgph1 mutant of M. oryzae. A. Organization of the NMR3 locus to show Pst I restriction sites and schematic representation showing mechanism of NMR3 gene deletion using split marker strategy. DNA was isolated from Δagl1Δgph1 and putative Δagl1Δgph1Δnmr3 transformants and digested with Pst I. The digested DNA was fractionated in a 0.8% agarose gel and transferred to Hybond-N. B. The membrane was probed with a 1 kb 5′ flanking DNA fragment to confirm Δagl1Δgph1Δnmr3 mutants on the basis of a restriction fragment length polymorphism. Transformant 10 was selected for further phenotypic analysis and hereafter named Δagl1Δgph1Δnmr3. WT = Guy11, 1–11 = putative Δagl1Δgph1Δnmr3 transformants and AG6 = Δagl1Δgph1. (PDF) [file ppat.1003604.s008.pdf]
