# Supplementary material for: Glycogen Metabolic Genes Are Involved in Trehalose-6-Phosphate Synthase-Mediated Regulation of Pathogenicity by the Rice Blast Fungus Magnaporthe oryzae
Source: PLoS Pathog. 2013 Oct 3;9(10):e1003604. doi: 10.1371/journal.ppat.1003604 (PMC3789717; doi:10.1371/journal.ppat.1003604)
Supplement: Figure S6 — Amino acid alignment of the predicted M. oryzae Gsn1 protein with reported glycogen synthases. The predicted M. oryzae GSN1 (MGG_07829.6) amino acid sequence was aligned with translated products of Homo sapiensf, Gys1 & Gys2, and Saccharomyces cerevisiae, Gsy1 (YFR015C) & Gsy2 (YLR258W). Sequences were identified from NCBI and SGD database (http://www.yeastgenome.org/), aligned using ClustalW (Thompson et al., 1994) and shaded using GeneDoc Version 2.6.002. Residues within black are identical among all listed proteins, conserved changes are shown in grey and those in white background do not show any similarity. Conserved putative serine or threonine residues that are candidates for post-translational phosphorylation are shown in blue. (DOC) [file ppat.1003604.s006.doc]

GYS1_H.sap 1 DNYFLVGPYTEQGVRTQVELLEAPTPA--------LKRTLDSMNSKGCKVYFGRWLIEGG
GYS2_H.sap 1 ENYFLIGPYFEHNMKTQVEQCEPVNDA--------VRRAVDAMNKHGCQVHFGRWLIEGS
MGG07289.6 1 DRYTLIGPLNHTSAAVEVEELEPKDPA--------LTATIQSMKDRGIGILYGRWLIEGA
YFR015C_Sc 1 DNYTLLGPLNKATYESEVEKLDWEDESIFPEELLPIQKTLMSMREKGVNFVYGNWLIEGA
YLR258W_Sc 1 DHYHLIGPLNKATYQNEVDILDWKKPEAFSDEMRPVQHALQTMESRGVHFVYGRWLIEGA


GYS1_H.sap 53 PLVVLLDVGASAWALERWKGELWDTCNIGVPWYDREANDAVLFGFLTTWFLGEFLAQSEE
GYS2_H.sap 53 PYVVLFDIGYSAWNLDRWKGDLWEACSVGIPYHDREANDMLIFGSLTAWFLKEV-TDHAD
MGG07289.6 53 PRVLLIDTKTAYKHLDEWKTDLWNVASIPSPPGDDETNEAIVFGYLVAWFLGEY-VCHEK
YFR015C_Sc 61 PRVILFELDSVRHFLNEWKADLWSLVGIPSPEHDHETNDAILLGYVVVWFLGEV-SKLDS
YLR258W_Sc 61 PKVILFDLDSVRGYSNEWKGDLWSLVGIPSPENDFETNDAILLGYTVAWFLGEV-AHLDS


GYS1_H.sap 113 KPHVVAHFHEWLAGVGLCLCRARRLPVATIFTTHATLLGRYLCA-GAVDFYNNLENFNVD
GYS2_H.sap 112 GKYVVAQFHEWQAGIGLILSRARKLPIATIFTTHATLLGRYLCA-ANIDFYNHLDKFNID
MGG07289.6 112 KKAVIAHFHEWLAGVGLPLCKKRRIDVTTIFTTHATLLGRYLCA-GSVDFYNNLQWFDVD
YFR015C_Sc 120 SHAIIGHFHEWLAGVALPLCRKKRIDVVTIFTTHATLLGRYLCAAGDVDFYNNLQYFDVD
YLR258W_Sc 120 QHAIVAHFHEWLAGVALPLCRKRRIDVVTIFTTHATLLGRYLCASGSFDFYNCLESVDVD


GYS1_H.sap 172 KEAGERQIYHRYCMERAAAHCAHVFTTVSQITAIEAQHLLKRKPDIVTPNGLNVKKFSAM
GYS2_H.sap 171 KEAGERQIYHRYCMERASVHCAHVFTTVSEITAIEAEHMLKRKPDVVTPNGLNVKKFSAV
MGG07289.6 171 AEAGKRGIYHRYCIERAAAHSCDVFTTVSHITAYESEHLLKRKPDGVLPNGLNVTKFAAM
YFR015C_Sc 180 QEAGKRGIYHRYCIERAAAHTADVFTTVSQITALEAEHLLKRKPDGILPNGLNVVKFQAV
YLR258W_Sc 180 HEAGRFGIYHRYCIERAAAHSADVFTTVSQITAFEAEHLLKRKPDGILPNGLNVIKFQAF


GYS1_H.sap 232 HEFQNLHAQSKARIQEFVRGHFYGHLDFNLDKTLYFFIAGRYEFSNKGADVFLEALARLN
GYS2_H.sap 231 HEFQNLHAMYKARIQDFVRGHFYGHLDFDLEKTLFLFIAGRYEFSNKGADIFLESLSRLN
MGG07289.6 231 HEFQNLHAQSKEKIHDFVRGHFYGHYDFEPENTLYFFTAGRYEFRNKGCDMFIESLARLN
YFR015C_Sc 240 HEFQNLHALKKDKINDFVRGHFHGCFDFDLDNTVYFFIAGRYEYKNKGADMFIESLARLN
YLR258W_Sc 240 HEFQNLHALKKEKINDFVRGHFHGCFDFDLDNTLYFFIAGRYEYKNKGADMFIEALARLN


GYS1_H.sap 292 YLLRVNGSEQTVVAFFIMPARTNNFNVETLKGQAVRKQLWDTANTVKEKFGRKLYESLLV
GYS2_H.sap 291 FLLRMHKSDITVMVFFIMPAKTNNFNVETLKGQAVRKQLWDVAHSVKEKFGKKLYDALLR
MGG07289.6 291 HRLKASGSKTTVVAFVIMPAQTSSLTVEALRGQAVVKSLRDTVDVIEKSIGRRIFERSLK
YFR015C_Sc 300 YRLKVSGSKKTVVAFLIMPAKTNSFTVEALKSQAIVKSLENTVNEVTASIGKRIFEHTMR
YLR258W_Sc 300 YRLKVSGSKKTVVAFIVMPAKNNSFTVEALKGQAEVRALENTVHEVTTSIGKRIFDHAIR


GYS1_H.sap 352 ----G---SLP-DMNKMLDKEDFTMMKRAIFATQRQ--SFPPVCTHNMLDDSSDPILTTI
GYS2_H.sap 351 ----G---EIP-DLNDILDRDDLTIMKRAIFSTQRQ--SLPPVTTHNMIDDSTDPILSTI
MGG07289.6 351 WHDGD---PLP-DEKELITSQDRVLLRRRLFAMKRH--GLPPIVTHNMVNDSEDPILNQI
YFR015C_Sc 360 YPHNGLESELPTNLDELLKSSEKVLLKKRVLALRRPYGELPPVVTHNMCDDANDPILNQI
YLR258W_Sc 360 YPHNGLTTELPTDLGELLKSSDKVMLKRRILALRRPEGQLPPIVTHNMVDDANDLILNKI


GYS1_H.sap 402 RRIGLFNSSADRVKVIFHPEFLSSTSPLLPVDYEEFVRGCHLGVFPSYYEPWGYTPAECT
GYS2_H.sap 401 RRIGLFNNRTDRVKVILHPEFLSSTSPLLPMDYEEFVRGCHLGVFPSYYEPWGYTPAECT
MGG07289.6 405 RRVQLFNHPTDRVKIVFHPEFLNSANPVLPLDYDDFVRGTHLGVFASYYEPWGYTPAECT
YFR015C_Sc 420 RHVRLFNDSSDRVKVIFHPEFLNANNPILGLDYDEFVRGCHLGVFPSYYEPWGYTPAECT
YLR258W_Sc 420 RQVQLFNSPSDRVKMIFHPEFLNANNPILGLDYDEFVRGCHLGVFPSYYEPWGYTPAECT


GYS1_H.sap 462 VMGIPSISTNLSGFGCFMEEHIA--DPSAYGIYILDRRFRSLDDSCSQLTSFLYSFCQQS
GYS2_H.sap 461 VMGIPSVTTNLSGFGCFMQEHVA--DPTAYGIYIVDRRFRSPDDSCNQLTKFLYGFCKQS
MGG07289.6 465 VMGVPSITTNLSGFGCYMEELIE--NSSDYGIYIVDRRTKGVDDSVNQLTNCMLEFCQKS
YFR015C_Sc 480 VMGVPSITTNVSGFGAYMEDLIETDQAKDYGIYIVDRRFKSPDESVEQLADYMEEFVNKT
YLR258W_Sc 480 VMGVPSITTNVSGFGAYMEDLIETNQAKDYGIYIVDRRFKAPDESVEQLVDYMEEFVKKT


GYS1_H.sap 520 RRQRIIQRNRTERLSDLLDWKYLGRYYMSARHMALSKAFPEHFT---------YEPNEAD
GYS2_H.sap 519 RRQRIIQRNRTERLSDLLDWRYLGRYYQHARHLTLSRAFPDKFH---------VELTSPP
MGG07289.6 523 RRQRINQRNRTERLSDLLDWKRMGLEYIKARQLALRRAYPNSFD--------GDDTADFM
YFR015C_Sc 540 RRQRINQRNRTERLSDLLDWKRMGLEYVKARQLGLRRAYPEQFKQLVGETISDANMNTLA
YLR258W_Sc 540 RRQRINQRNRTERLSDLLDWKRMGLEYVKARQLALRRGYPDQFRELVGEELNDSNMDALA

GYS1_H.sap 571 AAQGYRYPRPASVPPSPS-----LSRHSSPHQSEDEEDPRNGPLEEDGERYDEDEEAAKD
GYS2_H.sap 570 TTEGFKYPRPSSVPPSPS-----GSQASSPQSSDVED-------EVEDERYDEEEEAERD
MGG07289.6 575 NSSDLKISRPFSVPGSPR----DRAGMMTPGDFASLQEGREGLSTEDYVSWKLPEEEDPD
YFR015C_Sc 600 GGKKFKIARPLSVPGSPK-VRSNSTVYMTPGDLGTLQDAN---NADDYFNLSTNGAIDND
YLR258W_Sc 600 GGKKLKVARPLSVPGSPRDLRSNSTVYMTPGDLGTLQEVN---NADDYFSLGVNPAADDD


GYS1_H.sap 626 RRNIRAPEWPRRASCTSSTSGSKRNSVDTATSSSLSTPSEPLSPTSS--LGEERN
GYS2_H.sap 618 RLNIKSP------------------------FSLSHVPHGKKKL-----HGEYKN
MGG07289.6 631 EYTFPLT------------------------LGAKQRPSGPASPLDGVHLNGNGN
YFR015C_Sc 656 D---------------------------------DDNDTSAYY---------EDN
YLR258W_Sc 657 D-------------------------------------DGPYA---------DDS

**Figure S6.**
