# Supplementary material for: Glycogen Metabolic Genes Are Involved in Trehalose-6-Phosphate Synthase-Mediated Regulation of Pathogenicity by the Rice Blast Fungus Magnaporthe oryzae
Source: PLoS Pathog. 2013 Oct 3;9(10):e1003604. doi: 10.1371/journal.ppat.1003604 (PMC3789717; doi:10.1371/journal.ppat.1003604)
Supplement: Table S1 — Oligonucleotide primers used in this study. (DOCX) [file ppat.1003604.s009.docx]

**Table S1. Primers used in this study**

| **Name** | **Sequence (5'→3')** |
| --- | --- |
| Agl1-50.1 | CAGCGACAACAGTAACAGAAAAAAGTAAC |
| Agl1-NesF | AGCGATCGGCGATTGAGAGTG |
| Agl1-m13F | GTCGTGACTGGGAAAACCCTGGCGGTAGTACACCTCGTTGGAAATCATTGT |
| Agl1-m13R | TCCTGTGTGAAATTGTTATCCGCTCGCATGTGGGAGTACTATGTCGTAGAC |
| Agl1-30.1 | CATCCGTCGTTCCACCATTAACC |
| Agl1-NesR  M13F  M13R  IL-split | TGATTGGGGGTGCGGCG  GTCGTGACTGGGAAAACCCTGGCG  TCCTGTGTGAAATTGTTATCCGCT  AGTGGTGGTGACAGGAATCGA |
| LV-split  GSN1-50.1  GSN1-NesF  GSN1-m13F  GSN1-30.1  GSN1-NesR  GSN1-m13R  GSN1-5'  GSN1-3'  NMR3-50.1  NMR3-50.2  NMR3-m13F  NMR3-m13R  NMR3-30.1  NMR3-30.2  NMR3-5´  NMR3-3´  Agl1-Pro-U  Agl1-Fus-D  GFP-U  TrpC-D  Sur-U  Sur-D  Gph1-P-SacII-U  Gph1-Spe1-D  GFP-SpeI-U  TrpC-Cla1-D | TCATAGGCATCTCCCGGTCTT  GACAGACAGTTGAGTTTTGACC  CAAGCACCTCCCCAGATGAAA  GTCGTGACTGGGAAAACCCTGGCGAACGACATCAGTATTCTGTGGAA  TACCGTAGTCTTGTTGATGTGG  ACCCCCTCGTCGCTTCAGTA  TCCTGTGTGAAATTGTTATCCGCTAGATTGTCGCTTGGTTCCATGT  GCGCACAGAGTTGGCGGTAT  GTTACCGTTGCCGTTGAGATG  CTTCTCCCATTCGATGTTTGCA  GCGTCTGCATTCCCGTACAAA  GTCGTGACTGGGAAAACCCTGGCGAGTACCTCTTTGCCGCTTGTTT  TCCTGTGTGAAATTGTTATCCGCTTCCAACCAAGTACCACGTAGG  GGCTCAACATACCTCGATCAG  TTGGAGACTGATGGTGAGGCA  CATCCAAAACCCCAGCAACCA  CCAAGCCTCAAAGCCAAGCAT  AGCGACAACAGTAACAGAAAAA  GGTGAACAGCTCCTCGCCCTTGCTCACCATGTTCTTTTGCGACGACCCGTT  ATGGTGAGCAAGGGCGAGGA  TCACACAGGAAACAGCTATGACCATGATTAGCGACAACAGTAACAGAAAAA  AACTGTTGGGAAGGGCGATCGGTGCGGGCCCCAACGCCACAGTGCCCCA  CTGTTACTTTTTTCTGTTACTGTTGTCGCTGTGAGAGCATGCAATTCCCGT  AACCGCGGGCGCTGATCGTTATGAGGGTA  AAACTAGTCGCACCAACCTTGAGCGGC  AAACTAGTATGGTGAGCAAGGGCGAGGA  AAATCGATGATCCACTTAACGTTACTGAAATC |
